# Influence of Sociodemographic Variables on the Lifestyle of the Adult Population: A Multicenter Observational Study

**DOI:** 10.3390/healthcare13131564

**Published:** 2025-06-30

**Authors:** David García-García, Francisco Javier Pérez-Rivas, Tomás Gómez-Gascón, Ponte a 100 Clinical Investigators Group, Milagros Rico Blázquez, Marianela Bayón Cabeza, Susana Belmonte Cortés, Julia Domínguez-Bidagor, Jennifer Jiménez-González

**Affiliations:** 1 Grupo de Investigación UCM “Salud Pública-Estilos de Vida, Metodología Enfermera y Cuidados en el Entorno Comunitario”, Departamento de Enfermería, Facultad de Enfermería, Fisioterapia y Podología, Universidad Complutense de Madrid, 28040 Madrid, Spain; frjperez@ucm.es (F.J.P.-R.); or milagros.rico@salud.madrid.org (M.R.B.); jennjime@ucm.es (J.J.-G.); 2 Programa de Doctorado ‘Cuidados en Salud’, Facultad de Enfermería, Fisioterapia y Podología, Universidad Complutense de Madrid, 28040 Madrid, Spain; 3 Red de Investigación en Cronicidad, Atención Primaria y Promoción de la Salud—RICAPPS—(RICORS), Instituto de Salud Carlos III, 28029 Madrid, Spain; 4 Instituto de Investigación Sanitaria Hospital 12 de Octubre (Imas12), 28041 Madrid, Spain; 5 Fundación para la Investigación e Innovación Biosanitaria de Atención Primaria (FIIBAP), 28003 Madrid, Spain; 6 Unidad de Investigación de la Gerencia Asistencial de Atención Primaria, Servicio Madrileño de la Salud, 28035 Madrid, Spain; 7 Departamento de Enfermería, Facultad de Enfermería, Fisioterapia y Podología, Universidad Complutense de Madrid, Plaza Ramón y Cajal nº3, Ciudad Universitaria, 28040 Madrid, Spain; 8 Gregorio Marañon Health Research Institute, Madrid Health Service, 28007 Madrid, Spain; 9 Gerencia de Cuidados, Dirección General Asistencial, Servicio Madrileño de Salud, Consejería de Sanidad, Comunidad de Madrid, 28035 Madrid, Spain; marianela.bayon@salud.madrid.org; 10 Área de Nutrición y Estilos de Vida, Subdirección de Prevención y Promoción de la Salud, Dirección General de Salud Pública, Consejería de Sanidad, Comunidad de Madrid, 28002 Madrid, Spain; susana.belmonte@salud.madrid.org; 11 Unidad Técnica Promoción de Salud, Subdirección General Prevención y Promoción de Salud, Dirección General de Salud Pública, Comunidad de Madrid, 28002 Madrid, Spain; julia.dominguez@salud.madrid.org

**Keywords:** healthy lifestyle, primary health care, public health, socioeconomic factors

## Abstract

**Background/Objective:** The impact that lifestyle has on someone’s health has been widely proven. And the lifestyle can also be highly influenced by the sociodemographic background; however, there is less literature that focuses on this matter. Hence, the objective of the present study is to analyze the influence of sociodemographic variables on the lifestyle of the adult population. **Methods:** A cross-sectional multicenter study was conducted in 20 health centers of the Community of Madrid (Spain). A total of 365 participants were scheduled for nursing consultations and recruited through systematic probabilistic sampling. Lifestyle was assessed using the “Ponte a 100” questionnaire; based on the total score achieved by the participants, these were categorized into four groups depending on their need to adopt healthier lifestyle habits: minimal need (80–100 points), mild need (60–79 points), moderate need (40–59 points), and high need (<39 points). **Results**: Values ranged from 23 to 98 points in the Lifestyle Index (ISEV), with an average of 71.8 (SD = 14.6 points). Older individuals had better eating habits (β = −1.982), while younger individuals had better physical activity habits and a higher consumption of toxic substances. Men consumed more alcohol (β = −2.307) and felt happier with their lives, while women took more active breaks. Being a student was associated with higher levels of stress and anxiety, lower physical activity and leisure time, and higher alcohol consumption. Retirees had better emotional well-being indices (β = 0.999). Lower income levels were associated with occasional drug use and poorer emotional well-being. People with university degrees had better emotional states (β = 0.861) and better eating habits (β = 1.652) and engaged in more intense physical activity. Having no chronic diseases was related to being more physically active (β = 1.789). **Conclusions**: The study population generally presents a healthy lifestyle. The dimension of emotional well-being was the most influenced by sociodemographic factors. This study contributes to understanding the impact of socioeconomic variables on lifestyle.

## 1. Introduction

A healthy lifestyle can be understood as the set of choices and behaviors that promote physical, mental, and social balance. The impact of lifestyle on health has been evidenced in numerous studies, which link healthy lifestyle habits to a decrease in mortality and an increase in both quality of life and life expectancy free of disease or disability [1,2]. The cause–effect relationship between the adoption of healthy lifestyle behaviors and a reduced risk of death from more specific risk factors—such as tobacco use [3,4], alcohol consumption [5,6], physical activity and sedentary behavior [7,8], or diet [9,10]—has been widely demonstrated.

Importantly, these lifestyle determinants are not adopted in isolation but are shaped by sociodemographic and socioeconomic variables. For instance, dietary habits, including adherence to the Mediterranean diet, have been shown to vary by educational level, age, and cultural background [11,12]. Similarly, physical activity levels and sedentary behavior patterns are often influenced by employment status, urban environment, and gender roles [13,14]. Emotional well-being—another crucial lifestyle dimension—is closely linked to marital status, social support structures, and socioeconomic status, further underlining the multidimensional relationship between lifestyle and social context [15,16].

In order to analyze these relationships in a region-specific context, this study utilized the validated “Ponte a 100” questionnaire (Supplementary File S1), applied for the first time in the current study. This instrument was specifically developed for the adult population attending Primary Healthcare (PHC) services in Madrid and is designed to assess lifestyle through dimensions currently considered most relevant [17]: nutrition, physical activity and sedentary behavior, alcohol and tobacco or drug use, emotional well-being, and safety and unintentional injuries.

Unlike more generic tools such as the FANTASTIC questionnaire [18], MEDLIFE Index [19], HPLP-II [20], or the EVS questionnaire [21], “Ponte a 100” incorporates region-specific behaviors and urban lifestyle patterns relevant to Madrid’s population, such as local dietary trends and sedentary routines. Its use allows for a more nuanced understanding of how lifestyle behaviors vary across sociodemographic subgroups, offering insights that are directly applicable to targeted public health interventions.

Given the well-documented impact of age, sex, education, employment, and cultural context on individual lifestyle patterns [11,12,13,14,15], understanding these relationships is critical. The objective of this study was to analyze the lifestyles of an adult population receiving care in Primary Healthcare services in Madrid (Spain) and to assess the influence of sociodemographic variables on those lifestyles.

## 2. Materials and Methods

### 2.1. Design and Participants

A multicenter cross-sectional study was conducted with patients aged between 18 and 75 years who had scheduled nursing appointments at 20 Primary Healthcare Centers in the community of Madrid (Spain). All participants voluntarily agreed to take part and signed an informed consent form. Exclusion criteria included (1) patients receiving palliative care or with documented life expectancy of less than six months, as determined by clinical records and clinical assessment; (2) patients who were immobilized or institutionalized, as documented in the electronic health record; and (3) patients unable to provide informed consent or complete the questionnaire due to language barriers or severe cognitive impairment, identified through ICPC-2 coding (P70–Dementia) in the electronic health record. Data collection took place between February and July 2022. The sample of 365 participants was determined based on methodological recommendations for psychometric validation. Although there is no universal consensus, the literature suggests including between 5 and 10 participants per item [22,23]. Given that the “Ponte a 100” questionnaire includes 33 items, a minimum of 330 participants was required. Additionally, each clinical investigator was instructed to administer the questionnaire to 20 patients to enable inter- and intra-observer reliability analysis. This ensured both sufficient sample size and representation across sociodemographic subgroups [24]. Participants were recruited through systematic probabilistic sampling based on the schedule of nursing appointments. Each clinical investigator recruited 20 patients using a sequence of randomized numbers, selecting one patient per day within their appointment list.

### 2.2. Procedures

The nursing professionals who formed part of the clinical research team had prior experience in questionnaire validation and were trained to standardize data collection using an electronic notebook with the “Ponte a 100” tool. Each member of the clinical group was also provided with a folder containing all necessary information (study protocol, patient eligibility criteria, patient selection randomization sheet, patient information sheet, and informed consent form). All investigators signed an investigator commitment form prior to beginning the recruitment process.

Patients who were selected and voluntarily agreed to participate in the study underwent a face-to-face interview using the “Ponte a 100” questionnaire. Their responses were recorded in the electronic Case Report Form (CRF), and each participant was assigned a unique identification number to ensure anonymity. To ensure data quality, all participating nurses received specific training on standardized procedures for participant recruitment and questionnaire administration. The electronic data entry form included automated validation rules that prevented the recording of values outside the predefined range. Additionally, to minimize potential social desirability or interviewer bias, the questionnaire was previously validated in both self-administered and interviewer-administered formats, showing consistent scores across formats. The data were subsequently entered into a database created using Microsoft Office Excel 2013^®^.

### 2.3. Study Variables

The main variable, lifestyle, was measured using the “Ponte a 100” questionnaire. This tool was developed through a structured validation process including expert panel review, pilot testing, and psychometric evaluation [16,24]. It demonstrated high reproducibility, with intraclass correlation coefficients (ICCs) of 0.818 for self- versus nurse-administered formats, 1.000 for interobserver reliability, and 0.881 for intraobserver reliability. Internal consistency, assessed using Cronbach’s alpha, was modest (α = 0.41), a result consistent with other multidimensional lifestyle instruments due to the heterogeneity of domains assessed [18]. The questionnaire provides a maximum attainable score, defined as the Synthetic Lifestyle Index (ISEV), of 100 points (with a possible range from −35 to 100). This scoring system gives the tool its name: “Ponte a 100”. A higher score indicates a healthier lifestyle. The questionnaire includes a total of 33 items distributed across five dimensions: nutrition, physical activity and sedentary behavior, alcohol, tobacco and other drug use, emotional well-being, and safety and unintentional injuries. Each dimension has a maximum score of 25 points, and the total score is obtained by summing the scores of each individual dimension. However, certain dimensions are designed to allow for negative scoring. This serves a specific purpose: it helps to clearly identify and quantify particularly unhealthy behaviors that can significantly impact overall well-being. For example, excessive alcohol, tobacco, and other drug abuse may be scored negatively to reflect their detrimental effect on health. By incorporating negative values, the tool ensures that the overall score does not mask serious health risks in specific areas. These negative scores highlight the need for targeted interventions and emphasize that even if someone generally leads a healthy lifestyle, critical unhealthy behaviors should not be overlooked. [25] (Table 1).

Information was collected on sociodemographic variables such as sex (male, female), age (grouped as ≤35, 36–50, 51–65, and ≥66 years), nationality (Spanish, other nationality), and marital status (single, married or in a relationship, separated or divorced, widowed). Socioeconomic variables included level of education (illiterate or incomplete primary education, primary education, secondary education, high school and vocational training, university), occupation (employed by others, self-employed, unemployed, retired and pensioners, unpaid domestic work, student, unclassifiable), and monthly income (no income, ≤EUR 1000, EUR 1001–2500, >EUR 2501, prefers not to answer). Clinical variables related to health history were also considered, such as the presence of chronic illness (yes, no).

### 2.4. Statistical Analysis

A descriptive analysis of the study variables was performed. Quantitative variables were expressed as the mean and standard deviation (SD) when they followed a normal distribution; otherwise, they were presented as median and interquartile range. Normality was assessed using the Kolmogorov–Smirnov test. Qualitative variables were expressed as frequency distributions and percentages. To globally assess the need for lifestyle changes among patients, the ISEV variable was categorized into four groups: Minimal need (80–100 points), Mild need (60–79 points), Moderate need (40–59 points), and High need (<39 points). Given the small number of participants classified as having a “High need” for lifestyle intervention (n = 12; 3.3%), this category was merged with the “Moderate need” group to ensure sufficient statistical power for the analyses. The resulting combined group (“Moderate + High need”, score <59 points) may reduce the distinction between moderate and high intervention needs and should be interpreted with caution. To analyze the influence of sociodemographic and socioeconomic variables on lifestyle, an initial bivariate analysis was conducted using chi-square tests or Pearson correlation coefficients, as appropriate for qualitative or quantitative variables, respectively. No correction for multiple comparisons was applied to the bivariate analyses, as these were exploratory and intended to guide the construction of the multivariate regression model. Associations that showed statistically significant differences (*p* < 0.05) were included in a multiple linear regression analysis, considering the total score (ISEV) of the “Ponte a 100” questionnaire and each of its dimensions as dependent variables. Statistical analysis was performed using SPSS version 28.01.1 for Macintosh v20.0.

## 3. Results

### 3.1. Sample Description

The mean age of the sample was 54.9 years (SD = 15.1; range 17–75). Women represented 56.7% of the participants; 84.4% were of Spanish nationality; 57.3% were married or in a relationship; 39.5% had a university education; 42.5% were employed by others; 45.5% had a monthly income between EUR 1001 and EUR 2500; and 65.8% reported having a chronic illness (Table 2).

### 3.2. Lifestyle of the Study Population: Scores of the “Ponte a 100” Questionnaire

**Figure 1:**
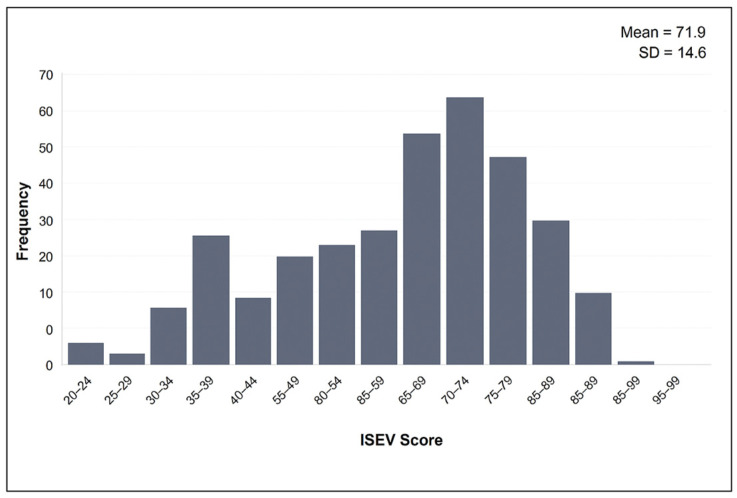
Distribution of ISEV scores among study participants.

The total scores obtained from the “Ponte a 100” questionnaire ranged from 23 to 98 points, with a mean score of 71.8 (SD = 14.6 points). The distribution of ISEV scores is shown in Figure 1. The mean score for its dimensions was 16.9 (SD = 3.6 points) for nutrition; 12.2 (SD = 5.7) for physical activity; 17.4 (SD = 10.3) for alcohol, tobacco, and other drugs; 15.9 (SD = 3.2) for emotional well-being; and 9.6 (SD = 0.9) for safety and unintentional injuries.

### 3.3. Influence of Sociodemographic Factors on the Overall Score (ISEV)

In the sample, 108 patients (30.1%) were classified as having a minimal need for lifestyle change, 180 (50.1%) as having a mild need, 59 (16.4%) as having a moderate need, and 12 (3.3%) as having a high need for change. Table 3 presents the results of these classifications based on the characteristics of the sample.

Regarding the factors that had a significant influence on the need for lifestyle intervention, it was identified that older individuals, those living with a partner, and retirees showed a lower need for intervention.

When performing the multiple linear regression model with the total score of the “Ponte a 100” tool as the dependent variable, it was observed that older individuals and those with higher levels of education had healthier lifestyles (Table 4).

### 3.4. Influence of Sociodemographic Factors on the Scores of the Dimensions of the “Ponte a 100” Questionnaire

For the nutrition dimension, in the bivariate analysis, significant differences were found for all sociodemographic variables except for the presence or absence of a chronic illness. These detailed associations are presented in Supplementary File S2: older individuals consumed more fruits, fish, and extra virgin olive oil (EVOO) and less meat and processed products. Higher income levels were associated with greater fish consumption. Spanish individuals consumed more fish and EVOO. Those with higher education levels consumed more raw nuts and less meat. Unemployed individuals, those doing unpaid domestic work (UDW), or self-employed individuals consumed fewer vegetables and fruits. In the multivariate analysis, being young had a negative impact, whereas being female and especially having a university education positively influenced this dimension (Table 5).

For the physical activity dimension, in the bivariate analysis, significant differences were found for all sociodemographic variables except for nationality. Supplementary File S3 includes the full bivariate results: younger individuals, those without chronic conditions, and those who were separated or divorced engaged in more moderate physical activity. Those with higher income levels, employed individuals, and those with higher education levels performed more intense physical activity (IPA). Women took more active breaks. In the multivariate analysis, being a student, illiterate, or having incomplete primary education had a negative impact, while not having a chronic illness positively influenced this dimension (Table 5).

For the alcohol, tobacco, and other drugs dimension, in the bivariate analysis, significant differences were found for all sociodemographic variables except for marital status. Supplementary File S4 presents the bivariate comparisons across sociodemographic groups: younger individuals, those without a university education, those without income, and students had higher drug consumption. Individuals aged 50–65 years, with chronic illnesses, and unemployed consumed more tobacco. Spanish individuals, students, and men consumed more alcohol. In the multivariate analysis, being male had a negative impact, whereas being retired or a pensioner had a positive influence (Table 5).

For the emotional well-being dimension, in the bivariate analysis, significant differences were found for all sociodemographic variables. Supplementary File S5 presents the full bivariate analysis: Married or partnered individuals and those with higher income levels felt more satisfied with themselves, happier with their lives, and more motivated to engage in new activities. Individuals over 65 years old, retirees, pensioners, and those of Spanish nationality reported feeling less stressed and having more leisure time. Men were generally happier with their lives. In the multivariate analysis, having no income, being single, and having a secondary education had a negative impact, while being retired or a pensioner and having a university education positively influenced this dimension (Table 5).

For the safety and unintentional injuries dimension, no significant differences were found for sociodemographic variables such as the presence or absence of chronic illness, nationality, education level, or sex. Supplementary File S6 includes the full bivariate data: Younger individuals followed traffic rules less. Married or partnered individuals drove less under the influence of alcohol and followed home safety recommendations more. Higher-income and self-employed individuals were less likely to follow traffic rules. Younger age and higher income levels had a negative impact (Table 5).

## 4. Discussion

The lifestyle of the surveyed population had an average score of 71.8 points, indicating that, in general, the population presents a fairly healthy lifestyle. This high overall score correlates well with the finding that 74% of the population in the Spanish National Health Survey rates their health as positive (good or very good) [26].

Older individuals, retirees, and those married or in a relationship exhibited a healthier lifestyle, with age and having a university education identified as positive predictors in the ISEV. Beyond educational level, many of these characteristics could be indirectly related to greater age, as older individuals tend to have higher resilience and a more traditional lifestyle associated with the Mediterranean diet. Existing literature already highlights the benefits of being married or in a relationship [27] and having a higher level of education [28].

Some of the lifestyle patterns observed may be influenced by contextual factors specific to the Madrid region. Access to free and universal public healthcare, a well-developed public transportation system, and adherence to the Mediterranean diet may support healthier lifestyle choices in certain population groups. Conversely, regional disparities in income, education, and housing conditions may help explain less favorable outcomes among individuals with a lower socioeconomic status. These contextual elements should be considered when designing targeted health promotion strategies.

When examining the specific dimensions of lifestyle, several patterns emerged across different population groups:

In the nutrition dimension, being young had a negative impact. In Spain, there has been an upward trend in the consumption of foods with low nutritional value and a decline in the consumption of fresh products like fruits, vegetables, and fish, especially among children [29]. This is accompanied by an increase in eating out [30], which is one of the main sources of processed food consumption [31]. Having a university education had a positive impact. The literature supports the idea that higher education levels are associated with healthier lifestyles [11]. Being female emerged as a positive predictor. The literature on gender differences in food consumption is mixed [28,30], but it is estimated that women in Spain consume more fruits and vegetables [32]. This could be influenced by the fact that usually women are often subjected from a young age to greater social pressure regarding body image, which may motivate more careful eating behaviors. This may have been influenced by the fact that women are traditionally more involved in meal planning and food preparation. However, some studies found that women may be more prone to eating disorders and modifying their nutrition habits due to emotional eating, which could be triggered by perceiving a situation as not manageable and stressful as a possible way of regulating negative emotions [33].

In the physical activity dimension, being a student had a negative impact, as this group engaged in the least moderate physical activity. This could be related to the findings in the emotional well-being dimension, where this group was observed to be more stressed and had less leisure time. Being illiterate or having incomplete primary education also emerged as a negative predictor, with higher education levels translating into a greater frequency of intense physical activity and active breaks. The literature suggests that individuals with lower education levels are the ones who most frequently fail to meet recommended physical activity levels [32]. Not having a chronic illness positively influenced this dimension, with this group engaging in more intense and moderate physical activity as well as muscle-strengthening exercises. This could be related to their better health status. These findings align with the results on emotional well-being, where individuals without chronic illnesses demonstrated greater motivation to undertake new activities [34].

In the alcohol, tobacco, and other drugs dimension, being male had a negative impact, with this group consuming more alcohol across all categories. This is supported by alcohol consumption data in Spain, which also indicates an increasing trend in binge drinking, especially among men across all age groups [35]. This also aligns with international studies, which also found that men consumed more alcoholic drinks than women. And that the amount of alcohol consumed was moderated by their anxiety levels [36]. Men appear to consume more alcohol and cannabis simultaneously due to enhancement and social motives such as improving mood and facilitating social situations [37]. Being retired or a pensioner emerged as a positive predictor, with this group consuming less alcohol, tobacco, and other drugs. These differences could largely be attributed to age, as younger individuals perceive a much lower risk [38].

In the emotional well-being dimension, having no income had a negative impact. The literature suggests that individuals in poverty and/or social exclusion are more susceptible to mental disorders, due to the barriers that a limited economic situation imposes [32]. Being single was also a negative predictor, with benefits observed for those who were accompanied (married or in a relationship), which is already supported by the literature [27]. Individuals with only compulsory education (primary/secondary) were the least satisfied with themselves, the least happy with their lives, and had the worst sleep, while those with a university education were more satisfied with themselves, happier with their lives, and more motivated to engage in new activities. These differences may be partly due to socioeconomic status, as individuals with lower education levels are generally at higher risk of poverty and social exclusion, and therefore greater vulnerability [39]. Lower income may impair emotional well-being through chronic stress and limited access to mental health resources, while university education likely fosters resilience by promoting socioeconomic stability. Potential mechanisms such as chronic stress, lack of social support, and reduced access to leisure or cultural activities could further explain these associations. Finally, retirees or pensioners were more frequently satisfied with their lives; never or almost never felt stressed, nervous, anxious, or irritable; and had more leisure time, likely due to their older age. This group is characterized by higher emotional resilience and greater tranquility due to fewer socioeconomic uncertainties in their lives.

In the safety and unintentional injuries dimension, being younger had a negative influence. No comparative studies were found that analyzed this dimension, but this finding might be related to lower perceived risk among younger individuals [40], although further research is needed to confirm this hypothesis. Higher income also had a negative impact, as these individuals were less likely to follow traffic rules. This could be explained by the lack of deterrence that an economic fine might cause for individuals who can afford higher expenses [38].

Despite its contributions, this study presents certain limitations. First, its cross-sectional design prevents establishing causal relationships between sociodemographic variables and lifestyle outcomes. Second, the sample was drawn from individuals attending PHC consultations, which may introduce selection bias and limit generalizability to the broader adult population. Third, data were collected through self-reported questionnaires in face-to-face interviews, which may be subject to social desirability or interviewer bias. To minimize this potential bias, interviewers were trained using a standardized protocol, and the instrument was previously validated in both self- and interviewer-administered formats, demonstrating high reproducibility.

Although some of the regression models yielded relatively low R^2^ values, this is not uncommon in studies involving complex human behaviors such as lifestyle choices. R^2^ (coefficient of determination) indicates the proportion of variance in the dependent variable explained by the independent variables. In behavioral and social science research, lower R^2^ values often reflect the multifactorial nature of lifestyle behaviors, which are influenced by a wide array of unmeasured factors, including personality traits, environmental conditions, cultural norms, and personal experiences. Despite the modest explanatory power of some models, the statistically significant associations identified remain meaningful. They highlight specific sociodemographic and socioeconomic variables—such as age, education, and employment status—that consistently influence lifestyle patterns. These insights are practically valuable for identifying vulnerable populations and designing targeted health promotion interventions, even if the models do not capture the full complexity of lifestyle determinants. In this context, low R^2^ values should not be interpreted as a limitation of the findings, but rather as a reminder of the inherent variability in human behavior and the importance of complementing quantitative analysis with broader, multi-dimensional approaches to health promotion.

Furthermore, although the questionnaire’s internal consistency was modest, this is expected for instruments that assess multiple heterogeneous lifestyle dimensions. Finally, the study was conducted in Madrid, a region with specific cultural and socioeconomic characteristics, which limits the extrapolation of findings to other contexts.

## 5. Conclusions

The study population generally exhibits a fairly healthy lifestyle. Older individuals, retirees, and those who are married or in a relationship have a healthier lifestyle, with age and having a university education identified as positive predictors in the ISEV. The emotional well-being dimension was the most influenced by sociodemographic and socioeconomic factors. Greater age, income level, retirement status, and higher educational level positively influenced the different dimensions of lifestyle. These findings enhance our understanding of how socioeconomic and demographic factors shape health-related behaviors and highlight the need to identify and support at-risk groups. Based on the results, targeted interventions should be prioritized for populations with lower educational levels, younger age, lower income, or unstable employment, as these groups are more vulnerable to adopting unhealthy lifestyle habits. Tailoring health promotion strategies in primary care to these high-risk profiles is essential to reduce health disparities and foster equitable, long-term improvements in population health. Policymakers may benefit from using the “Ponte a 100” questionnaire to identify population segments at greater risk and to inform the design of community-based or regional health promotion strategies. Clinicians, especially nurses in primary healthcare, can apply this tool in routine consultations as a rapid assessment instrument to guide lifestyle counseling and monitor change over time. Future research should build on these findings through longitudinal studies that assess changes in lifestyle behaviors over time, especially in response to contextual shifts such as retirement or changes in employment status. Additionally, the “Ponte a 100” tool offers promising potential for use in intervention studies, where it can serve both as a diagnostic instrument and an outcome measure to evaluate the effectiveness of health promotion programs in primary healthcare settings.

## Figures and Tables

**Table 1:** Distribution of Dimensions and Scores of the “Ponte a 100”.

Dimension	Items	Scores
Nutrition	11	0–25 points
Physical activity and Sedentarism	4	0–20 points
Alcohol	1	(−10)–10 points
Tobacco	1	(−15)–15 points
Other Drugs	1	(−10)–0 points
Emotional Well-being	10	0–20 points
Safety and Unintentional Injuries	5	0–10 points
Total (ISEV)	33	(−35)–100 points

Synthetic Index of Lifestyle (ISEV).

**Table 2:** Sample Description.

Variable		Frequency Distribution
Sex	Female	56.7%
	Male	40.8%
Nationality	Spanish	84.4%
	Other	13.2%
Marital Status	Single	24.9%
	Married	57.3%
	Separated or Divorced	8.8%
	Widowed	6.0%
Education level	Illiterate or incomplete primary education	1.9%
	Primary education	14.8%
	Secondary education	11.8%
	High School	29.6%
	University education	39.5%
Occupation	Employee	42.5%
	Self-employed	6.8%
	Retired/Pensioner	35.9%
	Unpaid domestic work	4.4%
	Students	3.0%
	Unclassified	0.5%
Monthly Income	No income	5.2%
	Less than or equal to EUR 1.000	21.6%
	From EUR 1.001 to EUR 2.500	45.5%
	More than EUR 2.500	8.8%
Chronic Illness	Yes	65.8%
	No	31.8%

**Table 3:** Analysis of the distribution of frequencies and percentages of the Synthetic Index of Lifestyles (ISEV) based on sociodemographic variables.

Variable		Need for Lifestyle Change (Total ISEV Score)		
		Great + Medium N (%fila)	Mild N (%fila)	Minimal N (%fila)
Sex	Female	35 (17.2)	93 (45.6)	76 (37.3)
	Male	29 (19.9)	70 (47.9)	47 (32.2)
	*p*	0.588		
Age	<46	24 (25.3)	48 (50.5)	23 (24.2)
	46–55	8 (13.8)	32 (55.2)	18 (31.0)
	56–65	25 (26.9)	37 (39.8)	31 (33.3)
	>65	7 (6.7)	46 (44.2)	51 (49.0)
	*p*	<0.001 **		
Nationality	Spanish	53 (17.5)	138 (45.7)	111 (36.8)
	Other	11 (22.9)	25 (52.1)	12 (25.0)
	*p*	0.267		
Marital Status	Single	24 (27.0)	42 (47.2)	23 (25.8)
	Married	24 (14.1)	93 (45.1)	84 (40.8)
	Separated, divorced, widowed	11 (20.4)	27 (50.0)	16 (29.6)
	*p*	0.033 **		
Education level	Primary studies	8 (13.6)	24 (40.7)	27 (45.8)
	Secondary studies	11 (25.6)	21 (48.8)	11 (25.6)
	Highschool	20 (18.7)	56 (52.3)	31 (29.0)
	University studies	25 (17.7)	62 (44.0)	54 (38.3)
	*p*	0.236		
Occupation	Working (employed/self-employed)	43 (24.0)	86 (48.0)	50 (27.9)
	Retired/pensioner	10 (7.9)	52 (41.3)	64 (50.8)
	Other (students, unemployed, unpaid domestic work)	10 (23.3)	25 (58.1)	8 (18.6)
	*p*	<0.001 **		
Monthly Income	No income	5 (26.3)	10 (52.6)	4 (21.1)
	Less than or equal to 1000EUR	10 (12.7)	44 (55.7)	25 (31.6)
	From 1001 to 2500EUR	29 (17.9)	72 (44.4)	61 (37.7)
	More than 2500EUR	3 (9.4)	17 (53.1)	12 (37.5)
	*p*	0.390		

Synthetic Index of Lifestyles (ISEV). Results with ** indicate statistical significance (*p* < 0.05).

**Table 4:** Multiple linear regression analysis of the dimensions of “Ponte a 100”.

Predictors	Beta (CI 95%)	*p*-Value
Total Score of “Ponte a 100” R 0.262/R^2^ 0.069/R^2^ adjusted 0.063/F14.241		
Constant	57.655 (51.624, 63.687)	<0.001
Education: University	3.491 (0.400, 6.583)	0.027
Age	0.234 (0.133, 0.334)	<0.001

**Table 5:** Multiple linear regression analysis of the “Ponte a 100” dimensions.

Predictors	Beta (CI 95%)	*p*-Value
Nutrition Dimension R 0.390/R^2^ 0.152/R^2^ adjusted 0.140/F12.943		
Constant	16.366 (15.662, 17.070)	<0.001
Education: University	1.652 (0.855, 2.449)	<0.001
Sex: Female	0.972 (0.188, 1.756)	0.015
Age: <35 years	−3.156 (−4.337, −1.975)	<0.001
Age: 36–50 years	−1.982 (−2.974, −0.989)	<0.001
Physical Activity Dimension R 0.270/R^2^ 0.073/R^2^ adjusted 0.063/F7.600		
Constant	11.773 (10.982, 12.565)	<0.001
Chronic disease: No	1.789 (0.412, 3.166)	0.011
Education: Illiterate or Incomplete Primary Education	−7.301 (−12.097, −2.165)	0.005
Occupation: Student	−5.647 (−9.208, −2.086)	0.002
Alcohol, Tobacco, and Other Drugs Dimension R 0.253/R^2^ 0.064/R^2^ adjusted 0.058/F10.022		
Constant	17.475 (15.857, 19.093)	<0.001
Occupation: Retired or Pensioner	4.679 (2.430, 6.928)	<0.001
Sex: Male	−2.307 (−4.494, −0.120)	0.039
Emotional Well-Being Dimension R 0.361/R^2^ 0.130/R^2^ adjusted 0.115/F8.592		
Constant	15.804 (15.139, 16.469)	<0.001
Occupation: Retired or Pensioner	0.999 (0.234, 1.764)	0.011
Education: University	0.861 (0.098, 1.624)	0.027
Education: Secondary Education	−1.885 (−3.057, −0.713)	0.002
Income: No Income	−1.526 (−2.990, −0.062)	0.041
Marital status: Single	−1.068 (−1.909, −0.227)	0.013
Safety and Unintentional Injuries Dimension R 0.308/R^2^ 0.095/R^2^ ajusted 0.086/F10.053		
Constant	9.760 (9.626, 9.893)	<0.001
Age: <35 years	−0.752 (−1.069, −0.436)	0.011
Income: >2501 euros	−0.428 (−0.763, −0.094)	0.012
Age: 36–50 years	−0.336 (−0.601, −0.071)	0.013

## Data Availability

The data presented in this study are available on request from the corresponding author.

## Supplementary Materials

The following supporting information can be downloaded at: https://www.mdpi.com/article/10.3390/healthcare13131564/s1, Supplementary File S1: The “PONTE A 100” Questionnaire; Supplementary File S2: Bivariate Analysis: Nutrition Dimension; Supplementary File S3: Bivariate Analysis: Physical Activity Dimension; Supplementary File S4: Bivariate Analysis: Alcohol, Tobacco, and Other Drugs Dimension; Supplementary File S5: Bivariate Analysis: Emotional Well-being Dimension; Supplementary File S6: Bivariate Analysis: Safety and Unintentional Injuries Dimension.

## References

[1] Li Y., Pan A., Wang D.D., Liu X., Dhana K., Franco O.H., Kaptoge S., Di Angelantonio E., Stampfer M., Willett W.C. (2018). Impact of Healthy Lifestyle Factors on Life Expectancies in the US Population. Circulation.

[2] Chudasama Y.V., Khunti K., Gillies C.L., Dhalwani N.N., Davies M.J., Yates T., Zaccardi F., Basu S. (2020). Healthy lifestyle and life expectancy in people with multimorbidity in the UK Biobank: A longitudinal cohort study. PLoS Med..

[3] Hackshaw A., Morris J.K., Boniface S., Tang J.-L., Milenković D. (2018). Low cigarette consumption and risk of coronary heart disease and stroke: Meta-analysis of 141 cohort studies in 55 study reports. BMJ.

[4] Gellert C., Schöttker B., Brenner H. (2012). Smoking and all-cause mortality in older people: Systematic review and meta-analysis. Arch. Intern. Med..

[5] Ortolá R., García-Esquinas E., López-García E., León-Muñoz L.M., Banegas J.R., Rodríguez-Artalejo F. (2018). Rodríguez-Artalejo, Alcohol consumption and all-cause mortality in older adults in Spain: An analysis accounting for the main methodological issues. Addiction.

[6] Wood A.M., Kaptoge S., Butterworth A.S., Willeit P., Warnakula S., Bolton T., Paige E., Paul D.S., Sweeting M., Burgess S. (2018). Risk thresholds for alcohol consumption: Combined analysis of individual-participant data for 599,912 current drinkers in 83 prospective studies. Lancet.

[7] Diaz K.M., Howard V.J., Hutto B., Colabianchi N., Vena J.E., Safford M.M., Blair S.N., Hooker S.P. (2017). Patterns of Sedentary Behavior and Mortality in U.S. Middle-Aged and Older Adults: A National Cohort Study. Ann. Intern. Med..

[8] Scott A.L., Hu W., Rangarajan S., Gasevic D., Leong D., Iqbal R., Casanova A., Swaminathan S., Anjana R.M., Kumar R. (2017). The effect of physical activity on mortality and cardiovascular disease in 130,000 people from 17 high-income, middle-income, and low-income countries: The PURE study. Lancet.

[9] Dehghan M., Mente A., Zhang X., Swaminathan S., Li W., Mohan V., Iqbal R., Kumar R., Wentzel-Viljoen E., Rosengren A. (2017). Associations of fats and carbohydrate intake with cardiovascular disease and mortality in 18 countries from five continents (PURE): A prospective cohort study. Lancet.

[10] Martínez-González M.A., Gea A., Ruiz-Canela M. (2019). The Mediterranean Diet and Cardiovascular Health. Circ. Res..

[11] Espinoza L.A., Sáurez K.R. (2020). Factores que influyen en el estilo de vida de los funcionarios de una universidad estatal de Costa Rica: Nivel educativo, estado civil y número de niños. UNED Res. J..

[12] Ministerio de Sanidad (2021). Acción Comunitaria para Ganar Salud. O Cómo Trabajar en Red para Mejorar las Condiciones de Vida.

[13] Pop L.-M., Iorga M., Șipoș L.-R., Iurcov R. (2021). Gender Differences in Healthy Lifestyle, Body Consciousness, and the Use of Social Networks among Medical Students. Medicina.

[14] Mishra G.D., Ball K., Dobson A.J., Byles J.E., Warner-Smith P. (2002). Warner-Smith, Which aspects of socio-economic status are related to health in mid-aged and older women?. Int. J. Behav. Med..

[15] Gil-Lacruz M., Gil-Lacruz A.I., Gracia-Pérez M.L. (2020). Health-related quality of life in young people: The importance of education. Health Qual. Life Outcomes.

[16] García-García D., Bazán M.J.A., Pérez-Rivas F.J. (2023). Correlation between Health and eHealth Literacy and a Healthy Lifestyle: A Cross-Sectional Study of Spanish Primary Healthcare Patients. Healthcare.

[17] Ministerio de Sanidad, Servicios Sociales e Igualdad (2014). Estrategia de Promoción de la Salud y Prevención en el SNS en el Marco del Abordaje de la Cronicidad en el SNS. Informes, Estudios e Investigación.

[18] Ramírez-Vélez R., Agredo R.A. (2012). Fiabilidad y validez del instrumento “Fantástico” para medir el estilo de vida en adultos colombianos. Rev. Salud Pública.

[19] Sotos-Prieto M. (2015). Validación de un Cuestionario para Medir los Hábitos de Estilo de Vida. Nutr. Hosp..

[20] Serrano-Fernández M.-J., Boada-Grau J., Gil-Ripoll C., Vigil-Colet A. (2016). Adaptación española de la escala HPLP-II con una muestra de empleados. Univ. Psychol..

[21] Reis F., Sá-Moura B., Guardado D., Couceiro P., Catarino L., Mota-Pinto A., Veríssimo M.T., Teixeira A.M., Ferreira P.L., Lima M.P. (2019). Development of a Healthy Lifestyle Assessment Toolkit for the General Public. Front. Med..

[22] Morales Vallejo P. (2011). Construcción de Cuestionarios y Escalas de Actitudes y Opiniones.

[23] Nunnally J., Bernstein I. (1994). Psychometric Theory.

[24] Jiménez-González J. (2024). Diseño, Validación y Aplicación del Cuestionario “Ponte a 100” para la Evaluación del Estilo de Vida en Población Adulta Atendida en Atención Primaria.

[25] Pérez-Rivas F.J., Jiménez-González J., Cabeza M.B., Cortés S.B., Díaz-Plaza M.d.D., Domínguez-Bidagor J., García-García D., Puente J.G., Gómez-Gascón T. (2023). Design and Content Validation using Expert Opinions of an Instrument Assessing the Lifestyle of Adults: The “PONTE A 100” Questionnaire. Healthcare.

[26] Ministerio de Sanidad Sanidad en Datos—Encuesta Nacional de Salud de España 2017. https://www.sanidad.gob.es/estadEstudios/estadisticas/encuestaNacional/encuesta2017.htm.

[27] Tumin D., Zheng H. (2018). Do the Health Benefits of Marriage Depend on the Likelihood of Marriage?. J. Marriage Fam..

[28] Unanua M.P., Fernández M.A., Simarro F.L., Llora T.S., Martínez I.P., Romero J.M. (2021). Adherencia a un estilo de vida saludable en pacientes con diabetes mellitus tipo 2 en España. Med. Fam. Semer..

[29] Ministerio de Sanidad (2022). Estrategia de Salud Pública 2022. Mejorando la Salud y el Bienestar de la Población. Plan de Re-Cuperación, Transformación y Resiliencia.

[30] Ministerio de Agricultura, Pesca y Alimentación (2022). Informe del Consumo Alimentario en España 2021.

[31] Instituto Nacional de Estadística (INE) (2016). Defunciones Según la Causa de Muerte 2014. http://www.ine.es/dynt3/inebase/index.htm?type=pcaxis&path=/t15/p417/a2014/&file=pcaxis.

[32] Ministerio de Sanidad (2024). Informe Anual del Sistema Nacional de Salud 2023.

[33] Hadar-Shoval D., Alon-Tirosh M., Asraf K., Tannous-Haddad L., Tzischinsky O. (2022). Lifestyle Changes, Emotional Eating, Gender, and Stress during COVID-19 Lockdown. Nutrients.

[34] Ministerio de Sanidad (2022). Actividad Física para la Salud y Reducción del Sedentarismo. Recomendaciones para la población. Estrategia de Promoción de la Salud y Prevención en el SNS.

[35] Sugerida C. (2021). Observatorio Español de las Drogas y las Adicciones. Monografía Alcohol 2021. Consumo y Consecuencias.

[36] Torres O.V., Estep J.C., Gwin M., Aramovich N.P., Thomas G., Villalta L. (2023). Distress symptoms and alcohol consumption: Anxiety differentially mediates drinking across gender. Front. Psychol..

[37] Tomko R.L., Gex K.S., Davis C.N., Schick M.R., Kirkland A.E., Squeglia L.M., Flanagan J.C., Gray K.M., McRae-Clark A.L. (2023). Sex and Gender Differences in Simultaneous Alcohol and Cannabis Use: A Narrative Review. Curr. Addict. Rep..

[38] Johnson R.J., McCaul K.D., Klein W.M.P. (2002). Klein, Risk Involvement and Risk Perception Among Adolescents and Young Adults. J. Behav. Med..

[39] Ministerio de Sanidad (2022). Informe Anual del Sistema Nacional de Salud. Informes, Estudios e Investigación.

[40] Vázquez I.A. (2020). Creencias sobre la salud y cambio de conducta. Manual de Psicología de la Salud.

