# Supplementary material for: Influence of Sociodemographic Variables on the Lifestyle of the Adult Population: A Multicenter Observational Study
Source: Healthcare (Basel). 2025 Jun 30;13(13):1564. doi: 10.3390/healthcare13131564 (PMC12250193; doi:10.3390/healthcare13131564)
Supplement: Supplementary file 1 [file healthcare-13-01564-s001.zip › Supplementary File S2_Nutrition Dimension.pdf]

Supplementary File S2: Analysis of the frequency and percentage distribution of responses to each item within the Nutrition dimension, according to sociodemographic variables.

Variable		Age				Sex		Nationality		Marital status				Level of Education					Occupation						Income				Chronic disease	
Nutrition		≤35	36-50	51-65	≥66	Female	Male	Other	Spanish	Single	Married In a relationship	Separated Divorced	Widowed	Illiterate or incomplete Primary Education	Primary Education	Secondary Education	High School or Further Education	University Education	Employed	Self-employed	Unemployed	Retired/Pensioner	Unpaid domestic work	Student	No Income	≤ a 1000	1001-2500	>2501	No	Yes
Vegetables	Does not consume	5 (11,6)	5 (7,5)	5 (5,7)	15 (9,5)	16 (7,7)	14 (9,4)	5 (10,4)	25 (8,1)	6 (6,6)	19 (9,1)	2 (6,3)	2 (9,1)	1 (14,3)	6 (11,1)	7 (16,3)	11 (10,2)	5 (3,5)	9 (5,8)	0 (0)	3 (18,8)	13 (9,9)	4 (25)	1 (9,1)	4 (21,1)	9 (11,4)	11 (6,6)	2 (6,3)	5 (4,3)	25 (10,4)
	1 serving	19 (44,2)	31 (46,3)	34 (38,6)	63 (39,9)	78 (37,7)	69 (46,3)	24 (50)	123 (39,9)	39 (42,9)	84 (40,2)	14 (43,8)	10 (45,5)	2 (28,6)	24 (44,4)	19 (44,2)	49 (45,4)	53 (36,8)	70 (45,2)	16 (64)	5 (31,3)	46 (35,1)	4 (25)	4 (36,4)	7 (36,8)	34 (43)	67 (40,4)	18 (56,3)	45 (38,8)	102 (42,5)
	More than 1 serving	19 (44,2)	31 (46,3)	49 (55,7)	80 (50,6)	113 (54,6)	66 (44,3)	19 (39,6)	160 (51,9)	46 (50,5)	106 (50,7)	16 (50)	10 (45,5)	4 (57,1)	24 (44,4)	17 (39,5)	48 (44,4)	86 (59,7)	76 (49)	9 (36)	8 (50)	72 (55)	8 (50)	6 (54,5)	8 (42,1)	36 (45,6)	88 (53)	12 (37,5)	66 (56,9)	113 (47,1)
	P	0,762				0,159		0,281		0,985				0,063					0,039**						0,203				0,074	
Fruits	Does not consume	7 (16,3)	9 (13,4)	10 (11,4)	11 (7)	21 (10,1)	16 (10,7)	6 (12,5)	31 (10,1)	13 (14,3)	18 (8,6)	3 (9,4)	3 (13,6)	1 (14,3)	5 (9,3)	6 (14)	12 (11,1)	13 (9)	16 (10,3)	3 (12)	4 (25)	9 (6,9)	4 (25)	1 (9,1)	3 (15,8)	11 (13,9)	14 (8,4)	3 (9,4)	11 (9,5)	26 (10,8)
	1-2 servings	24 (55,8)	27 (40,3)	35 (39,8)	51 (32,3)	76 (36,7)	61 (40,9)	19 (39,6)	118 (38,3)	39 (42,9)	76 (36,4)	13 (40,6)	7 (31,8)	4 (57,1)	18 (33,3)	15 (34,9)	43 (39,8)	57 (39,6)	71 (45,8)	13 (52)	4 (25)	38 (29)	4 (25)	6 (54,5)	8 (42,1)	30 (38)	66 (39,8)	16 (50)	50 (43,1)	87 (36,3)
	More than 2 servings	12 (27,9)	31 (46,3)	43 (48,9)	96 (60,8)	110 (53,1)	72 (48,3)	23 (47,9)	159 (51,6)	39 (42,9)	115 (55)	16 (50)	12 (54,5)	2 (28,6)	31 (57,4)	22 (51,2)	53 (49,1)	74 (51,4)	68 (43,9)	9 (36)	8 (50)	84 (64,1)	8 (50)	4 (36,4)	8 (42,1)	38 (48,1)	86 (51,8)	13 (40,6)	55 (47,4)	127 (52,9)
	P	0,010**				0,662		0,833		0,535				0,903					0,017**						0,690				0,460	
Whole grains	Does not consume	17 (39,5)	28 (41,8)	48 (54,5)	86 (54,4)	92 (44,4)	87 (58,4)	17 (35,4)	162 (52,6)	37 (40,7)	111 (53,1)	17 (53,1)	13 (59,1)	4 (57,1)	30 (55,6)	24 (55,8)	54 (50)	67 (46,5)	73 (47,1)	16 (64)	10 (62,5)	69 (52,7)	7 (43,8)	3 (27,3)	10 (52,6)	41 (51,9)	89 (53,6)	18 (56,3)	53 (45,7)	126 (52,5)
	1 serving	13 (30,2)	21 (31,3)	25 (28,4)	50 (31,6)	71 (34,3)	38 (25,5)	22 (45,8)	87 (28,2)	32 (35,2)	58 (27,8)	12 (37,5)	6 (27,3)	2 (28,6)	15 (27,8)	11 (25,6)	34 (31,5)	47 (32,6)	46 (29,7)	8 (32)	3 (18,8)	43 (32,8)	6 (37,5)	3 (27,3)	6 (31,6)	29 (36,7)	41 (24,7)	9 (28,1)	36 (31)	73 (30,4)
	2 servings	8 (18,6)	10 (14,9)	9 (10,2)	10 (6,3)	25 (12,1)	12 (8,1)	5 (10,4)	32 (10,4)	11 (12,1)	25 (12)	0 (0)	1 (4,5)	1 (14,3)	2 (3,7)	4 (9,3)	12 (11,1)	18 (12,5)	20 (12,9)	1 (4)	1 (6,3)	8 (6,1)	2 (12,5)	4 (36,4)	3 (15,8)	4 (5,1)	23 (13,9)	3 (9,4)	12 (10,3)	25 (10,4)
	More than 2 servings	5 (11,6)	8 (11,9)	6 (6,8)	12 (7,6)	19 (9,2)	12 (8,1)	4 (8,3)	27 (8,8)	11 (12,1)	15 (7,2)	3 (9,4)	2 (9,1)	0 (0)	7 (13)	4 (9,3)	8 (7,4)	12 (8,3)	16 (10,3)	0 (0)	2 (12,5)	11 (8,4)	1 (6,3)	1 (9,1)	0 (0)	5 (6,3)	13 (7,8)	2 (6,3)	15 (12,9)	16 (6,7)
	P	0,246				0,71		0,084		0,298				0,865					0,190						0,474				0,235	
Nuts	Does not consume	15 (34,9)	21 (31,3)	35 (39,8)	45 (28,5)	64 (30,9)	52 (34,9)	22 (45,8)	94 (30,5)	34 (37,4)	67 (32,1)	9 (28,1)	5 (22,7)	2 (28,6)	28 (51,9)	12 (27,9)	40 (37)	34 (23,6)	52 (33,5)	6 (24)	7 (43,8)	40 (30,5)	6 (37,5)	4 (36,4)	7 (36,8)	32 (40,5)	53 (31,9)	8 (25)	32 (27,6)	84 (35)
	1-3 servings, raw or roasted	18 (41,9)	28 (41,8)	33 (37,5)	64 (40,5)	86 (41,5)	57 (38,3)	16 (33,3)	127 (41,2)	33 (36,3)	86 (41,1)	13 (40,6)	11 (50)	3 (42,9)	17 (31,5)	25 (58,1)	38 (35,2)	60 (41,7)	66 (42,6)	12 (48)	4 (25)	53 (40,5)	3 (18,8)	4 (36,4)	4 (21,1)	27 (34,2)	71 (42,8)	13 (40,6)	49 (42,2)	94 (39,2)
	4-7 servings, raw	10 (23,3)	18 (26,9)	20 (22,7)	49 (31)	57 (27,5)	40 (26,8)	10 (20,8)	87 (28,2)	24 (26,4)	56 (26,8)	10 (31,3)	6 (27,3)	2 (28,6)	9 (16,7)	6 (14)	30 (27,8)	50 (34,7)	37 (23,9)	7 (28)	5 (31,3)	38 (29)	7 (43,8)	3 (27,3)	8 (42,1)	20 (25,3)	42 (25,3)	11 (34,4)	35 (30,2)	62 (25,8)
	P	0,638				0,715		0,107		0,851				0,002**					0,767						0,307				0,360	
Oil	Does not consume	9 (20,9)	12 (17,9)	11 (12,5)	15 (9,5)	24 (11,6)	23 (15,4)	14 (29,2)	33 (10,7)	19 (20,9)	24 (11,5)	2 (6,3)	2 (9,1)	1 (14,3)	8 (14,8)	6 (14)	15 (13,9)	17 (11,8)	23 (14,8)	7 (28)	0 (0)	13 (9,9)	2 (12,5)	2 (18,2)	2 (10,5)	13 (16,5)	25 (15,1)	3 (9,4)	19 (16,4)	28 (11,7)
	Consumes olive oil	8 (18,6)	14 (20,9)	8 (9,1)	18 (11,4)	30 (14,5)	18 (12,1)	12 (25)	36 (11,7)	19 (20,9)	21 (10)	4 (12,5)	4 (18,2)	2 (28,6)	6 (11,1)	12 (27,9)	14 (13)	14 (9,7)	22 (14,2)	4 (16)	2 (12,5)	16 (12,2)	2 (12,5)	1 (9,1)	3 (15,8)	13 (16,5)	19 (11,4)	3 (9,4)	15 (12,9)	33 (13,8)
	Consumes extra virgin olive oil	26 (60,5)	41 (61,2)	69 (78,4)	125 (79,1)	153 (73,9)	108 (72,5)	22 (45,8)	239 (77,6)	53 (58,2)	164 (78,5)	26 (81,3)	16 (72,7)	4 (57,1)	40 (74,1)	25 (58,1)	79 (73,1)	113 (78,5)	110 (71)	14 (56)	14 (87,5)	102 (77,9)	12 (75)	8 (72,7)	14 (73,7)	53 (67,1)	122 (73,5)	26 (81,3)	82 (70,7)	179 (74,6)
	P	0,037**				0,505		0,000**		0,016**				0,147					0,391						0,784				0,469	
Legumes	Does not consume	6 (14)	7 (10,4)	6 (6,8)	12 (7,6)	22 (10,6)	9 (6)	7 (14,6)	24 (7,8)	9 (9,9)	16 (7,7)	1 (3,1)	5 (22,7)	2 (28,6)	6 (11,1)	3 (7)	5 (4,6)	15 (10,4)	14 (9)	4 (16)	0 (0)	10 (7,6)	1 (6,3)	2 (18,2)	3 (15,8)	9 (11,4)	10 (6)	2 (6,3)	11 (9,5)	20 (8,3)
	1-2 servings	25 (58,1)	36 (53,7)	51 (58)	94 (59,5)	133 (64,3)	73 (49)	23 (47,9)	183 (59,4)	52 (57,1)	120 (57,4)	22 (68,8)	11 (50)	2 (28,6)	25 (46,3)	24 (55,8)	68 (63)	87 (60,4)	98 (63,2)	13 (52)	7 (43,8)	74 (56,5)	10 (62,5)	3 (27,3)	9 (47,4)	43 (54,4)	97 (58,4)	25 (78,1)	67 (57,8)	139 (57,9)

	More than 2 servings	12 (27,9)	24 (35,8)	31 (35,2)	52 (32,9)	52 (25,1)	67 (45)	18 (37,5)	101 (32,8)	30 (33)	73 (34,9)	9 (28,1)	6 (27,3)	3 (42,9)	23 (42,6)	16 (37,2)	35 (32,4)	42 (29,2)	43 (27,7)	8 (32)	9 (56,3)	47 (35,9)	5 (31,3)	6 (54,5)	7 (36,8)	27 (34,2)	59 (35,5)	5 (15,6)	38 (32,8)	81 (33,8)
	P	0,802				<0,001**		0,181		0,234				0,164					0,314						0,146				0,931	
Fish	Does not consume	7 (16,3)	8 (11,9)	4 (4,5)	9 (5,7)	14 (6,8)	14 (9,4)	5 (10,4)	23 (7,5)	12 (13,2)	12 (5,7)	3 (9,4)	1 (4,5)	0 (0)	4 (7,4)	2 (4,7)	10 (9,3)	12 (8,3)	11 (7,1)	3 (12)	3 (18,8)	6 (4,6)	2 (12,5)	3 (27,3)	3 (15,8)	10 (12,7)	9 (5,4)	1 (3,1)	9 (7,8)	19 (7,9)
	1-2 servings	19 (44,2)	35 (52,2)	37 (42)	65 (41,1)	91 (44)	65 (43,6)	28 (58,3)	128 (41,6)	42 (46,2)	90 (43,1)	15 (46,9)	8 (36,4)	4 (57,1)	23 (42,6)	22 (51,2)	53 (49,1)	54 (37,5)	71 (45,8)	11 (44)	6 (37,5)	56 (42,7)	7 (43,8)	4 (36,4)	7 (36,8)	39 (49,4)	76 (45,8)	9 (28,1)	52 (44,8)	104 (43,4)
	More than 2 servings	17 (39,5)	24 (35,8)	47 (53,4)	84 (53,2)	102 (49,3)	70 (47)	15 (31,3)	157 (51)	37 (40,7)	107 (51,2)	14 (43,8)	13 (59,1)	3 (42,9)	27 (50)	19 (44,2)	45 (41,7)	78 (54,2)	73 (47,1)	11 (44)	7 (43,8)	69 (52,7)	7 (43,8)	4 (36,4)	9 (47,4)	30 (38)	8 1 (48,8)	22 (68,8)	55 (47,4)	117 (48,8)
	P	0,044**				0,650		0,039**		0,280				0,592					0,420						0,039**				0,965	
Meat	More than 4 servings	8 (18,6)	11 (16,4)	5 (5,7)	7 (4,4)	14 (6,8)	17 (11,4)	7 (14,6)	24 (7,8)	8 (8,8)	19 (9,1)	2 (6,3)	2 (9,1)	1 (14,3)	3 (5,6)	10 (23,3)	12 (11,1)	5 (3,5)	18 (11,6)	0 (0)	3 (18,8)	7 (5,3)	2 (12,5)	1 (9,1)	2 (10,5)	8 (10,1)	13 (7,8)	2 (6,3)	12 (10,3)	19 (7,9)
	3-4 servings	11 (25,6)	24 (35,8)	19 (21,6)	21 (13,3)	40 (19,3)	35 (23,5)	12 (25)	63 (20,5)	25 (27,5)	42 (20,1)	6 (18,8)	2 (9,1)	1 (14,3)	10 (18,5)	6 (14)	24 (22,2)	34 (23,6)	40 (25,8)	8 (32)	3 (18,8)	18 (13,7)	3 (18,8)	3 (27,3)	5 (26,3)	17 (21,5)	37 (22,3)	9 (28,1)	28 (24,1)	47 (19,6)
	Less than 3 servings	24 (55,8)	32 (47,8)	64 (72,7)	130 (82,3)	153 (73,9)	97 (65,1)	29 (60,4)	221 (71,8)	58 (63,7)	148 (70,8)	24 (75)	18 (81,8)	5 (71,4)	41 (75,9)	27 (62,8)	72 (66,7)	105 (72,9)	97 (62,6)	17 (68)	10 (62,5)	106 (80,9)	11 (68,8)	7 (63,6)	12 (63,2)	54 (68,4)	116 (69,9)	21 (65,6)	76 (65,5)	174 (72,5)
	P	0,000**				0,148		0,186		0,574				0,013**					0,096						0,969				0,398	
Processed/Ultra-processed foods	More than 4 servings	12 (27,9)	11 (16,4)	9 (10,2)	11 (7)	24 (11,6)	19 (12,8)	6 (12,5)	37 (12,1)	14 (15,4)	23 (11)	2 (6,3)	4 (18,2)	0 (0)	4 (7,4)	12 (27,9)	18 (16,7)	9 (6,3)	24 (15,5)	5 (20)	1 (6,3)	9 (6,9)	3 (18,8)	1 (9,1)	4 (21,1)	9 (11,4)	22 (13,3)	3 (9,4)	17 (14,7)	26 (10,9)
	3-4 servings	10 (23,3)	11 (16,4)	4 (4,5)	13 (8,3)	17 (8,2)	21 (14,2)	8 (16,7)	30 (9,8)	16 (17,6)	17 (8,1)	4 (12,5)	1 (4,5)	0 (0)	2 (3,7)	2 (4,7)	13 (12)	21 (14,6)	19 (12,3)	3 (12)	2 (12,5)	10 (7,7)	1 (6,3)	3 (27,3)	2 (10,5)	5 (6,3)	19 (11,5)	4 (12,5)	18 (15,5)	20 (8,4)
	Less than 3 servings	21 (48,8)	45 (67,2)	75 (85,2)	133 (84,7)	166 (80,2)	108 (73)	34 (70,8)	240 (78,2)	61 (67)	169 (80,9)	26 (81,3)	17 (77,3)	6 (100)	48 (88,9)	29 (67,4)	77 (71,3)	114 (79,2)	112 (72,3)	17 (68)	13 (81,3)	111 (85,4)	12 (75)	7 (63,6)	13 (68,4)	65 (82,3)	124 (75,2)	25 (78,1)	81 (69,8)	193 (80,8)
	P	**0,000****				0,168		0,343		0,109				0,001**					0,288						0,729				0,054	
Carbonated/Sugary/Sweetened beverages	Sugary	6 (14)	13 (19,4)	8 (9,1)	14 (8,9)	17 (8,2)	24 (16,2)	9 (18,8)	32 (10,4)	10 (11)	27 (12,9)	3 (9,4)	1 (4,5)	1 (16,7)	4 (7,4)	11 (25,6)	14 (13)	11 (7,6)	23 (14,8)	1 (4)	0 (0)	12 (9,2)	3 (18,8)	2 (18,2)	2 (10,5)	11 (13,9)	23 (13,9)	0 (0)	13 (11,2)	28 (11,7)
	Sweetened	7 (16,3)	16 (23,9)	22 (25)	23 (14,6)	42 (20,3)	26 (17,6)	5 (10,4)	63 (20,5)	17 (18,7)	38 (18,2)	6 (18,8)	7 (31,8)	0 (0)	13 (24,1)	10 (23,3)	23 (21,3)	22 (15,3)	33 (21,3)	5 (20)	5 (31,3)	19 (14,6)	3 (18,8)	3 (27,3)	6 (31,6)	15 (19)	30 (18,2)	3 (9,4)	21 (18,1)	47 (19,7)
	Does not consume	30 (69,8)	38 (56,7)	58 (65,9)	120 (76,4)	148 (71,5)	98 (66,2)	34 (70,8)	212 (69,1)	64 (70,3)	144 (68,9)	23 (71,9)	14 (63,6)	5 (83,3)	37 (68,5)	22 (51,2)	71 (65,7)	111 (77,1)	99 (63,9)	19 (76)	11 (68,8)	99 (76,2)	10 (62,5)	6 (54,5)	11 (57,9)	53 (67,1)	112 (67,9)	29 (90,6)	82 (70,7)	164 (68,6)
	P	0,057				0,065		0,094		0,740				0,024**					0,391						0,111				0,921	
Water	Does not consume	1 (2,3)	1 (1,5)	0 (0)	3 (1,9)	4 (1,9)	1 (0,7)	0 (0)	5 (1,6)	2 (2,2)	2 (1)	1 (3,1)	0 (0)	0 (0)	0 (0)	0 (0)	3 (2,8)	2 (1,4)	0 (0)	0 (0)	1 (6,3)	3 (2,3)	0 (0)	1 (9,1)	2 (10,5)	1 (1,3)	1 (0,6)	0 (0)	1 (0,9)	4 (1,7)
	Less than 4 glasses	8 (18,6)	6 (9)	13 (14,8)	22 (14)	31 (15)	18 (12,2)	5 (10,4)	44 (14,3)	10 (11)	29 (13,9)	2 (6,3)	7 (31,8)	2 (33,3)	6 (11,1)	6 (14)	14 (13)	21 (14,6)	24 (15,5)	4 (16)	2 (12,5)	13 (10)	3 (18,8)	3 (27,3)	4 (21,1)	6 (7,6)	22 (13,3)	4 (12,5)	20 (17,2)	29 (12,1)
	More than 4 glasses	34 (79,1)	60 (89,6)	75 (85,2)	132 (84,1)	172 (83,1)	129 (87,2)	43 (89,6)	258 (84)	79 (86,8)	178 (85,2)	29 (90,6)	15 (68,2)	4 (66,7)	48 (88,9)	37 (86)	91 (84,3)	121 (84)	131 (84,5)	21 (84)	13 (81,3)	114 (87,7)	13 (81,3)	7 (63,6)	13 (68,4)	72 (91,1)	142 (86,1)	28 (87,5)	95 (81,9)	206 (86,2)
	P	0,668				0,442		0,498		0,138				0,724					0,220						0,011**				0,366	

N (column %). Results marked with ** are statistically significant (p<0.05).
