# Supplementary material for: Influence of Sociodemographic Variables on the Lifestyle of the Adult Population: A Multicenter Observational Study
Source: Healthcare (Basel). 2025 Jun 30;13(13):1564. doi: 10.3390/healthcare13131564 (PMC12250193; doi:10.3390/healthcare13131564)
Supplement: Supplementary file 1 [file healthcare-13-01564-s001.zip › Supplementary File S3_ Physical Activity Dimension.pdf]

Supplementary File S3: Analysis of the frequency and percentage distribution of responses to each item within the Physical Activity dimension, according to sociodemographic variables.

**Variable**		**Age**				**Sex**		**Nationality**		**Marital status**				**Level of Education**					**Occupation**						**Income**				**Chronic disease**	
**Physical Activity**		≤35	36-50	51-65	≥66	Female	Male	Other	Spanish	Single	Married In a relationship	Separated Divorced	Widowed	Illiterate or incomplete Primary Education	Primary Education	Secondary Education	High School or Further Education	University Education	Employed	Self-employed	Unemployed	Retired/Pensioner	Unpaid domestic work	Student	No Income	≤ a 1000	1001-2500	>2501	No	Yes
**Intense Physical Activity**	3 o más veces	7 (16,3)	16 (23,9)	15 (17)	19 (12)	45 (18,5)	15 (12,3)	9 (18,8)	48 (15,6)	13 (14,3)	40 (19,1)	2 (6,3)	2 (9,1)	0 (0)	4 (7,4)	5 (11,6)	15 (13,9)	33 (22,9)	33 (21,3)	5 (20)	3 (18,8)	14 (10,7)	0 (0)	2 (18,2)	1 (5,3)	12 (15,2)	29 (17,5)	10 (31,3)	30 (25,9)	27 (11,3)
	1-2 veces	12 (27,9)	15 (22,4)	13 (14,8)	16 (10,1)	41 (16,9)	16 (13,1)	7 (14,6)	49 (15,9)	22 (24,2)	25 (12)	6 (18,8)	3 (13,6)	1 (14,3)	4 (7,4)	4 (9,3)	17 (15,7)	30 (20,8)	34 (21,9)	5 (20)	2 (12,5)	12 (9,2)	1 (6,3)	1 (9,1)	2 (10,5)	8 (10,1)	28 (16,9)	8 (25)	23 (19,8)	33 (13,8)
	Nunca	24 (55,8)	36 (53,7)	60 (68,2)	123 (77,8)	157 (64,6)	91 (74,6)	32 (66,7)	211 (68,5)	56 (61,5)	144 (68,9)	24 (75)	17 (77,3)	6 (85,7)	46 (85,2)	34 (79,1)	76 (70,4)	81 (56,3)	88 (56,8)	15 (16)	11 (68,8)	105 (80,2)	15 (93,8)	8 (72,7)	16 (84,2)	59 (74,7)	109 (65,7)	14 (43,8)	63 (54,3)	180 (75)
	P	0,005**				0,147		0,849		0,080				0,007**					0,010**						0,039**				0,000**	
**Moderate Physical Activity**	5 o más veces	13 (30,2)	32 (47,8)	40 (45,5)	95 (60,1)	128 (52,7)	55 (45,1)	29 (60,4)	151 (49)	43 (47,3)	109 (52,2)	18 (56,3)	9 (40,9)	2 (28,6)	34 (63)	21 (48,8)	56 (51,9)	67 (46,5)	65 (41,9)	13 (52)	8 (50)	83 (63,4)	8 (50)	2 (18,2)	7 (36,8)	43 (54,4)	8 1 (48,8)	15 (46,9)	56 (48,3)	124 (51,7)
	3-4 veces	11 (25,6)	17 (25,4)	14 (15,9)	20 (12,7)	42 (17,3)	23 (18,9)	6 (12,5)	56 (18,2)	19 (20,9)	33 (15,8)	9 (28,1)	1 (4,5)	1 (14,3)	7 (13)	6 (14)	18 (16,7)	30 (20,8)	35 (22,6)	2 (8)	1 (6,3)	20 (15,3)	2 (12,5)	2 (18,2)	3 (15,8)	12 (15,2)	33 (19,9)	4 (12,5)	27 (23,3)	35 (14,6)
	1-2 veces	14 (32,6)	12 (17,9)	17 (19,3)	20 (12,7)	40 (16,5)	25 (20,5)	9 (18,8)	54 (17,5)	22 (24,2)	31 (14,8)	4 (12,5)	6 (27,3)	1 (14,3)	4 (7,4)	9 (20,9)	18 (16,7)	31 (21,5)	37 (23,9)	4 (16)	6 (37,5)	9 (6,9)	2 (12,5)	4 (36,4)	3 (15,8)	15 (19)	27 (16,3)	8 (25)	26 (22,4)	37 (15,4)
	Nunca	5 (11,6)	6 (9)	17 (19,3)	23 (14,6)	33 (13,6)	19 (15,6)	4 (8,3)	47 (15,3)	7 (7,7)	36 (17,2)	1 (3,1)	6 (27,3)	3 (42,9)	9 (16,7)	7 (16,3)	16 (14,8)	16 (11,1)	18 (11,6)	6 (24)	1 (6,3)	19 (14,5)	4 (25)	3 (27,3)	6 (31,6)	9 (11,4)	25 (15,1)	5 (15,6)	7 (6)	44 (18,3)
	P	0,004**				0,570		0,353		0,016**				0,245					0,002**						0,566				0,003**	
**Muscle strenghtening**	2 o más veces	17 (39,5)	13 (19,4)	18 (20,5)	29 (18,4)	58 (23,9)	20 (16,4)	14 (29,2)	63 (20,5)	22 (24,2)	42 (20,1)	9 (28,1)	4 (18,2)	0 (0)	11 (20,4)	6 (14)	23 (21,3)	37 (25,7)	39 (25,2)	5 (20)	4 (25)	24 (18,3)	3 (18,8)	2 (18,2)	4 (21,1)	17 (21,5)	36 (21,7)	6 (18,8)	37 (31,9)	40 (16,7)
	1 vez	4 (9,3)	6 (9)	7 (8)	10 (6,3)	16 (6,6)	11 (9)	3 (6,3)	24 (7,8)	12 (13,2)	14 (6,7)	1 (3,1)	0 (0)	0 (0)	4 (7,4)	5 (11,6)	9 (8,3)	9 (6,3)	11 (7,1)	0 (0)	2 (12,5)	11 (8,4)	0 (0)	2 (18,2)	1 (5,3)	9 (11,4)	10 (6)	2 (6,3)	7 (6)	20 (8,3)
	Nunca	22 (51,2)	48 (71,6)	63 (71,6)	119 (75,3)	169 (69,5)	91 (74,6)	31 (64,6)	221 (71,8)	57 (62,6)	153 (73,2)	22 (68,8)	18 (81,8)	7 (100)	39 (72,2)	32 (74,4)	76 (70,4)	98 (68,1)	105 (67,7)	20 (80)	10 (62,5)	96 (73,3)	13 (81,3)	7 (63,6)	14 (73,7)	53 (67,1)	120 (72,3)	24 (75)	72 (62,1)	180 (75)
	P	0,093				0,216		0,389		0,167				0,558					0,327						0,846				0,005**	
**Active rest**	Siempre o casi siempre	16 (37,2)	33 (49,3)	50 (56,8)	79 (50)	137 (56,4)	44 (36,1)	27 (56,3)	151 (49)	38 (41,8)	110 (52,6)	17 (53,1)	11 (50)	2 (28,6)	28 (51,9)	16 (37,2)	50 (46,3)	82 (56,9)	79 (51)	11 (44)	11 (68,8)	66 (50,4)	6 (37,5)	5 (45,5)	9 (47,4)	34 (43)	84 (50,6)	20 (62,5)	62 (53,4)	116 (48,3)
	Algunas veces	16 (37,2)	12 (17,9)	21 (23,9)	42 (26,6)	58 (23,9)	37 (30,3)	11 (22,9)	80 (26)	26 (28,6)	54 (25,8)	5 (15,9)	6 (27,3)	1 (14,3)	19 (35,2)	10 (23,3)	28 (25,9)	33 (22,9)	34 (21,9)	7 (28)	3 (18,8)	41 (31,3)	2 (12,5)	3 (27,3)	3 (15,8)	24 (30,4)	37 (22,3)	7 (21,9)	28 (24,1)	63 (26,3)
	Nunca o casi nunca	11 (25,6)	22 (32,8)	17 (19,3)	37 (23,4)	48 (19,8)	41 (33,6)	10 (20,8)	77 (25)	27 (29,7)	45 (21,5)	10 (31,3)	5 (22,7)	4 (57,1)	7 (13)	17 (39,5)	30 (27,8)	29 (20,1)	42 (27,1)	7 (28)	2 (12,5)	24 (18,3)	8 (50)	3 (27,3)	7 (36,8)	21 (26,6)	45 (27,1)	5 (15,6)	26 (22,4)	61 (25,4)
	P	0,164				0,001**		0,644		0,478				0,020**					0,209						0,404				0,659	

N (column %). Results marked with ** are statistically significant ($p<0.05$).
