# Supplementary material for: Influence of Sociodemographic Variables on the Lifestyle of the Adult Population: A Multicenter Observational Study
Source: Healthcare (Basel). 2025 Jun 30;13(13):1564. doi: 10.3390/healthcare13131564 (PMC12250193; doi:10.3390/healthcare13131564)
Supplement: Supplementary file 1 [file healthcare-13-01564-s001.zip › Supplementary File S4_ Alcohol, Tobacco, and Other Drugs Dimension.pdf]

Supplementary File S4: Analysis of the frequency and percentage distribution of responses to each item within the Alcohol, Tobacco, and Other Drugs dimension, according to sociodemographic variables.

**Variable**		**Age**				**Sex**		**Nationality**		**Marital status**				**Level of Education**					**Occupation**						**Income**				**Chronic disease**	
**Alcohol, Tobacco and Other Drugs**		≤35	36-50	51-65	≥66	Female	Male	Other	Spanish	Single	Married In a relationship	Separated Divorced	Widowed	Illiterate or incomplete Primary Education	Primary Education	Secondary Education	High School or Further Education	University Education	Employed	Self-employed	Unemployed	Retired/Pensioner	Unpaid domestic work	Student	No Income	≤ a 1000	1001-2500	>2501	No	Yes
**Alcohol SDUs/week**	No consumption	20 (46,5)	30 (44,8)	46 (52,3)	96 (60,8)	128 (61,8)	64 (43)	20 (41,7)	172 (55,8)	41 (45,1)	118 (56,5)	15 (46,9)	17 (77,3)	5 (71,4)	40 (74,1)	27 (62,8)	57 (52,8)	63 (43,8)	80 (51,6)	8 (32)	7 (43,8)	79 (60,3)	12 (75)	5 (45,5)	10 (52,6)	54 (68,4)	85 (51,2)	13 (40,6)	50 (43,1)	142 (59,2)
	Women: up to 7 SDUs/week Men: up to 14 SDUs/week	15 (34,9)	31 (46,3)	29 (33)	45 (28,5)	59 (28,5)	61 (40,9)	16 (33,3)	104 (33,8)	34 (37,4)	68 (32,5)	13 (40,6)	4 (18,2)	2 (28,6)	8 (14,8)	12 (27,9)	38 (35,2)	60 (41,7)	59 (38,1)	10 (40)	7 (43,8)	39 (29,8)	3 (18,8)	2 (18,2)	6 (31,6)	18 (22,8)	59 (35,5)	14 (43,8)	50 (43,1)	70 (29,2)
	Women: 8-12 SDUs/week Men: 15-21 SDUs/week	6 (14)	4 (6)	6 (6,8)	12 (7,6)	12 (5,8)	16 (10,7)	6 (12,5)	22 (7,1)	10 (11)	14 (6,7)	3 (9,4)	1 (4,5)	0 (0)	2 (3,7)	3 (7)	9 (8,3)	14 (9,7)	9 (5,8)	3 (12)	2 (12,5)	1 0 (7,6)	0 (0)	3 (27,3)	2 (10,5)	5 (6,3)	14 (8,4)	3 (9,4)	11 (9,5)	17 (7,1)
	Women: 13-17 SDUs/week Men: 22-28 SDUs/week	1 (2,3)	0 (0)	3 (3,4)	4 (2,5)	4 (1,9)	4 (2,7)	2 (4,2)	6 (1,9)	3 (3,3)	5 (2,4)	0 (0)	0 (0)	0 (0)	3 (5,6)	0 (0)	3 (2,8)	2 (1,4)	3 (1,9)	1 (4)	0 (0)	3 (2,3)	0 (0)	1 (9,1)	1 (5,3)	1 (1,3)	4 (2,4)	1 (3,1)	2 (1,7)	6 (2,5)
	Women: >17 SDUs/week Men: >28 SDUs/week	1 (2,3)	2 (3)	4 (4,5)	1 (0,6)	4 (1,9)	4 (2,7)	4 (8,3)	4 (1,3)	3 (3,3)	4 (1,9)	1 (3,1)	0 (0)	0 (0)	1 (1,9)	1 (2,3)	1 (0,9)	5 (3,5)	4 (2,6)	3 (12)	0 (0)	0 (0)	1 (6,3)	0 (0)	0 (0)	1 (1,3)	4 (2,4)	1 (3,1)	3 (2,6)	5 (2,1)
	P	0,193				0,012**		0,011**		0,506				0,059					0,014**						0,544				0,058	
**Do you smoke?**	No	31 (72,1)	49 (73,1)	68 (77,3)	137 (86,7)	164 (79,2)	121 (81,2)	40 (83,3)	245 (79,5)	66 (72,5)	178 (85,2)	22 (68,8)	17 (77,3)	7 (100)	43 (79,6)	32 (74,4)	87 (80,6)	116 (80,6)	114 (73,5)	20 (80)	8 (50)	120 (91,6)	13 (81,3)	9 (81,8)	15 (78,9)	66 (83,5)	137 (82,5)	29 (90,6)	90 (77,6)	195 (81,3)
	Yes, occasionally	3 (7)	2 (3)	2 (2,3)	2 (1,3)	7 (3,4)	2 (1,3)	1 (2,1)	8 (2,6)	5 (5,5)	4 (1,9)	0 (0)	0 (0)	0 (0)	1 (1,9)	2 (4,7)	2 (1,9)	4 (2,8)	5 (3,2)	1 (4)	2 (12,5)	1 (0,8)	0 (0)	0 (0)	1 (5,4)	0 (0)	5 (3)	0 (0)	5 (4,3)	4 (1,7)
	Yes, 1-10 cigarettes/day 1 hookah/day	8 (18,6)	11 (16,4)	7 (8)	13 (8,2)	26 (12,6)	13 (8,7)	5 (10,4)	34 (11)	14 (15,4)	16 (7,7)	6 (18,8)	3 (13,6)	0 (0)	4 (7,4)	6 (14)	17 (15,7)	12 (8,3)	20 (12,9)	3 (12)	4 (25)	8 (6,1)	2 (12,5)	2 (18,2)	3 (15,8)	11 (13,9)	13 (7,8)	2 (6,3)	18 (15,5)	21 (8,8)
	Yes, 11-20 cigarettes/day 2 hookahs/day	1 (2,3)	4 (6)	8 (9,1)	6 (3,8)	8 (3,9)	11 (7,4)	2 (4,2)	17 (5,5)	5 (5,5)	8 (8,3)	4 (12,5)	2 (9,1)	0 (0)	3 (5,6)	3 (7)	1 (0,9)	12 (8,3)	13 (8,4)	1 (4)	2 (12,5)	2 (1,5)	0 (0)	0 (0)	0 (0)	2 (2,5)	9 (5,4)	1 (3,1)	3 (2,6)	16 (6,7)
	Yes, more than 20 cigarettes/day 3 hookahs/day	0 (0)	1 (1,5)	3 (3,4)	0 (0)	2 (1)	2 (1,3)	0 (0)	4 (1,3)	1 (1,1)	3 (1,4)	0 (0)	0 (0)	0 (0)	3 (5,6)	0 (0)	1 (0,9)	0 (0)	3 (1,9)	0 (0)	0 (0)	0 (0)	1 (6,3)	0 (0)	0 (0)	0 (0)	2 (1,2)	0 (0)	0 (0)	4 (1,7)
	P	0,034**				0,314		0,925		0,129				0,064					0,006**						0,525				0,042**	
**Do you use any other substances or drugs?**	No	38 (88,4)	67 (100)	87 (98,9)	158 (100)	205 (99)	145 (97,3)	47 (97,9)	303 (98,4)	86 (94,5)	208 (99,5)	32 (100)	22 (100)	7 (100)	51 (94,4)	43 (100)	105 (97,2)	144 (100)	153 (98,7)	25 (100)	16 (100)	130 (99,2)	15 (93,8)	9 (81,8)	16 (84,2)	78 (98,7)	165 (99,4)	32 (100)	112 (96,6)	238 (99,2)
	Yes, occasionally	4 (9,3)	0 (0)	0 (0)	0 (0)	1 (0,5)	3 (2)	1 (2,1)	3 (1)	4 (4,4)	0 (0)	0 (0)	0 (0)	0 (0)	1 (1,9)	0 (0)	3 (2,8)	0 (0)	2 (1,3)	0 (0)	0 (0)	0 (0)	0 (0)	2 (18,2)	3 (15,8)	0 (0)	1 (0,6)	0 (0)	3 (2,6)	1 (0,4)
	Yes, daily	1 (2,3)	0 (0)	1 (1,1)	0 (0)	1 (0,5)	1 (0,7)	0 (0)	2 (0,6)	1 (1,1)	1 (0.5)	0 (0)	0 (0)	0 (0)	2 (3,7)	0 (0)	0 (0)	0 (0)	0 (0)	0 (0)	0 (0)	1 (0,8)	1 (6,3)	0 (0)	0 (0)	1 (1,3)	0 (0)	0 (0)	1 (0,9)	1 (0,4)
	P	0,000**				0,389		0,681		0,051				0,037**					0,000**						0,000**				0,165	

N (column %). Results marked with ** are statistically significant (p<0.05).
