# Supplementary material for: Influence of Sociodemographic Variables on the Lifestyle of the Adult Population: A Multicenter Observational Study
Source: Healthcare (Basel). 2025 Jun 30;13(13):1564. doi: 10.3390/healthcare13131564 (PMC12250193; doi:10.3390/healthcare13131564)
Supplement: Supplementary file 1 [file healthcare-13-01564-s001.zip › Supplementary File S5_ Emotional Well-being Dimension.pdf]

Supplementary File S5: Analysis of the frequency and percentage distribution of responses to each item within the Emotional Well-being dimension, according to sociodemographic variables.

**Variable**		**Age**				**Sex**		**Nationality**		**Marital status**				**Level of Education**					**Occupation**						**Income**				**Chronic disease**	
**Emotional Well-being**		≤35	36-50	51-65	≥66	Female	Male	Other	Spanish	Single	Married In a relationship	Separated Divorced	Widowed	Illiterate or incomplete Primary Education	Primary Education	Secondary Education	High School or Further Education	University Education	Employed	Self-employed	Unemployed	Retired/Pensioner	Unpaid domestic work	Student	No Income	≤ a 1000	1001-2500	>2501	No	Yes
**Satisfied with myself**	Always or almost always	23 (53,5)	45 (67,2)	56 (63,6)	110 (69,6)	129 (62,3)	105 (70,5)	28 (58,3)	206 (66,9)	48 (52,7)	149 (71,3)	19 (59,4)	17 (77,3)	5 (71,4)	24 (44,4)	28 (65,1)	71 (65,7)	106 (73,6)	104 (67,1)	16 (64)	11 (68,8)	91 (69,5)	7 (43,8)	4 (36,4)	8 (42,1)	48 (60,8)	114 (68,7)	26 (81,3)	80 (69)	154 (64,2)
	Sometimes	20 (46,5)	16 (23,9)	27 (30,7)	42 (26,6)	69 (33,3)	36 (24,2)	16 (33,3)	89 (28,9)	36 (39,6)	53 (25,4)	12 (37,5)	4 (18,2)	2 (28,6)	27 (50)	11 (25,6)	32 (29,6)	33 (22,9)	41 (26,5)	8 (32)	4 (25)	35 (26,7)	9 (56,3)	7 (63,6)	11 (57,9)	27 (34,2)	44 (26,5)	5 (15,6)	33 (28,4)	72 (30)
	Never or almost never	0 (0)	6 (9)	5 (5,7)	6 (3,8)	9 (4,3)	8 (5,4)	4 (8,3)	13 (4,2)	7 (7,7)	7 (3,3)	1 (3,1)	1 (4,5)	0 (0)	3 (5,6)	4 (9,3)	5 (4,6)	5 (3,5)	10 (6,5)	1 (4)	1 (6,3)	5 (3,8)	0 (0)	0 (0)	0 (0)	4 (5,1)	8 (4,8)	1 (3,1)	3 (2,6)	14 (5,8)
	P	0,066				0,171		0,331		0,048**				0,022**					0,230						0,047**				0,356	
**Content with the life I lead**	Always or almost always	25 (58,1)	35 (52,2)	56 (63,6)	109 (69)	120 (58)	105 (70,5)	24 (50)	201 (65,3)	49 (53,8)	149 (71,3)	16 (50)	10 (45,5)	2 (28,6)	35 (64,8)	22 (51,2)	65 (60,2)	101 (70,1)	92 (59,4)	19 (76)	7 (43,8)	91 (69,5)	7 (43,8)	7 (63,6)	10 (52,6)	46 (58,2)	111 (66,9)	26 (81,3)	71 (61,2)	154 (64,2)
	Sometimes	13 (30,2)	29 (43,3)	24 (27,3)	41 (25,9)	73 (35,3)	34 (22,8)	21 (43,8)	86 (27,9)	31 (34,1)	49 (23,4)	16 (50)	10 (45,5)	5 (71,4)	14 (25,9)	12 (27,9)	40 (37)	36 (25)	52 (33,5)	2 (8)	9 (56,3)	33 (25,2)	8 (50)	3 (27,3)	6 (31,6)	28 (35,4)	47 (28,3)	4 (12,5)	38 (32,8)	69 (28,7)
	Never or almost never	5 (11,6)	3 (4,5)	8 (9,1)	8 (5,1)	14 (6,8)	10 (6,7)	3 (6,3)	21 (6,8)	11 (12,1)	11 (5,3)	0 (0)	2 (9,1)	0 (0)	5 (9,3)	9 (20,9)	3 (2,8)	7 (4,9)	11 (7,1)	4 (16)	0 (0)	7 (5,3)	1 (6,3)	1 (9,1)	3 (15,8)	5 (6,3)	8 (4,8)	2 (6,3)	7 (6)	17 (7,1)
	P	0,098				0,037**		0,082		0,001**				0,000**					0,048**						0,124				0,721	
**Cheerful and in a good mood**	Always or almost always	21 (48,8)	38 (56,7)	59 (67)	98 (62)	120 (58)	96 (64,4)	25 (52,1)	191 (62)	45 (49,5)	135 (64,6)	20 (62,5)	15 (68,2)	4 (57,1)	28 (51,9)	25 (58,1)	64 (59,3)	95 (66)	102 (65,8)	15 (60)	7 (43,8)	81 (61,8)	6 (37,5)	4 (36,4)	6 (31,6)	41 (51,9)	112 (67,5)	23 (71,9)	63 (54,3)	153 (63,7)
	Sometimes	22 (51,2)	25 (37,3)	28 (31,8)	56 (35,4)	82 (39,6)	49 (32,9)	22 (45,8)	109 (35,4)	43 (47,3)	68 (32,5)	12 (37,5)	7 (31,8)	3 (42,9)	23 (42,6)	16 (37,2)	41 (38)	48 (33,3)	50 (32,3)	9 (36)	8 (50)	47 (35,9)	9 (56,3)	7 (63,6)	12 (63,2)	34 (43)	52 (31,3)	8 (25)	51 (44)	80 (33,3)
	Never or almost never	0 (0)	4 (6)	1 (1,1)	4 (2,5)	5 (2,4)	4 (2,7)	1 (2,1)	8 (2,6)	3 (3,3)	6 (2,9)	0 (0)	0 (0)	0 (0)	3 (5,6)	2 (4,7)	3 (2,8)	1 (0,7)	3 (1,9)	1 (4)	1 (6,3)	3 (2,3)	1 (6,3)	0 (0)	1 (5,3)	4 (5,1)	2 (1,2)	1 (3,1)	2 (1,7)	7 (2,9)
	P	0,134				0,430		0,377		0,224				0,497					0,439						0,013**				0,136	
**Stressed, nervous, anxious, irritable…**	Always or almost always	9 (20,9)	15 (22,4)	19 (21,6)	10 (6,3)	32 (15,5)	21 (14,1)	12 (25)	41 (13,3)	20 (22)	26 (12,4)	3 (9,4)	3 (13,6)	0 (0)	5 (9,3)	12 (27,9)	17 (15,7)	19 (13,2)	30 (19,4)	7 (28)	3 (18,8)	9 (6,9)	1 (6,3)	3 (27,3)	4 (21,1)	10 (12,7)	26 (15,7)	4 (12,5)	15 (12,9)	38 (15,8)
	Sometimes	25 (58,1)	40 (59,7)	49 (55,7)	86 (54,4)	125 (60,4)	75 (50,3)	28 (58,3)	172 (55,8)	51 (56)	117 (56)	18 (56,3)	14 (63,6)	5 (71,4)	33 (61,1)	20 (46,5)	62 (57,4)	80 (55,6)	90 (58,1)	15 (60)	10 (62,5)	67 (51,1)	10 (62,5)	7 (63,6)	13 (68,4)	51 (64,6)	79 (47,6)	20 (62,5)	68 (58,6)	132 (55)
	Never or almost never	9 (20,9)	12 (17,9)	20 (22,7)	62 (39,2)	50 (24,2)	53 (35,6)	8 (16,7)	95 (30,8)	20 (22)	66 (31,6)	11 (34,4)	5 (22,7)	2 (28,6)	16 (29,6)	11 (25,6)	29 (26,9)	45 (31,3)	35 (22,6)	3 (12)	3 (18,8)	55 (42)	5 (31,3)	1 (9,1)	2 (10,5)	18 (22,8)	61 (36,7)	8 (25)	33 (28,4)	70 (29,2)
	P	0,000**				0,062		0,034**		0,276				0,318					0,004**						0,077				0,727	
**Motivated to engage in new activities**	Always or almost always	27 (62,8)	49 (73,1)	65 (73,9)	96 (60,8)	134 (64,7)	103 (69,1)	31 (64,6)	206 (66,9)	59 (64,8)	143 (68,4)	21 (65,6)	12 (54,5)	3 (42,9)	29 (53,7)	23 (53,5)	74 (68,5)	108 (75)	111 (71,6)	19 (76)	11 (68,8)	82 (62,6)	7 (43,8)	5 (45,5)	8 (42,1)	56 (70,9)	115 (69,3)	25 (78,1)	87 (75)	150 (62,5)
	Sometimes	12 (27,9)	13 (19,4)	16 (18,2)	41 (25,9)	48 (23,2)	34 (22,8)	11 (22,9)	71 (23,1)	24 (26,4)	47 (22,5)	9 (28,1)	2 (9,1)	2 (28,6)	17 (31,5)	9 (20,9)	25 (23,1)	29 (20,1)	30 (19,4)	2 (8)	4 (25)	34 (26)	6 (37,5)	6 (54,5)	10 (52,6)	13 (16,5)	39 (23,5)	4 (12,5)	23 (19,8)	59 (24,6)
	Never or almost never	4 (9,3)	5 (7,5)	7 (8)	21 (13,3)	25 (12,1)	12 (8,1)	6 (12,5)	31 (10,1)	8 (8,8)	19 (9,1)	2 (6,3)	8 (36,4)	2 (28,6)	8 (14,8)	11 (25,6)	9 (8,3)	7 (4,9)	14 (9)	4 (16)	1 (6,3)	15 (11,5)	3 (18,8)	0 (0)	1 (5,3)	10 (12,7)	12 (7,2)	3 (9,4)	6 (5,2)	31 (12,9)
	P	0,347				0,448		0,874		0,005**				0,002**					0,117						0,020**				0,028**	
**Able to overcome difficulties**	Always or almost always	27 (62,8)	52 (77,6)	70 (79,5)	128 (81)	158 (76,3)	119 (79,9)	39 (81,3)	238 (77,3)	61 (67)	169 (80,9)	27 (84,4)	18 (81,8)	6 (85,7)	37 (68,5)	33 (76,7)	81 (75)	120 (83,3)	120 (77,4)	19 (76)	10 (62,5)	109 (83,2)	10 (62,5)	8 (72,7)	10 (52,6)	61 (77,2)	134 (80,7)	26 (81,3)	91 (78,4)	186 (77,5)
	Sometimes	16 (37,2)	13 (19,4)	15 (17)	28 (17,7)	45 (21,7)	27 (18,1)	8 (16,7)	64 (20,8)	27 (29,7)	36 (17,2)	5 (15,6)	4 (18,2)	0 (0)	16 (29,6)	8 (18,6)	25 (23,1)	23 (16)	32 (20,6)	5 (20)	5 (31,3)	21 (16)	5 (31,3)	3 (27,3)	7 (36,8)	18 (22,8)	28 (16,9)	5 (15,6)	23 (19,8)	49 (20,4)
	Never or almost never	0 (0)	2 (3)	3 (3,4)	2 (1,3)	4 (1,9)	3 (2)	1 (2,1)	6 (1,9)	3 (3,3)	4 (1,9)	0 (0)	0 (0)	1 (14,3)	1 (1,9)	2 (4,7)	2 (1,9)	1 (0,7)	3 (1,9)	1 (4)	1 (6,3)	1 (0,8)	1 (6,3)	0 (0)	2 (10,5)	0 (0)	4 (2,4)	1 (3,1)	2 (1,7)	5 (2,1)
	P	0,085				0,704		0,804		0,174				0,057					0,558						0,039**				0,964	
**Supportive environment**	Always or almost always	41 (95,3)	55 (82,1)	77 (87,5)	131 (82,9)	183 (88,4)	121 (81,2)	38 (79,2)	266 (86,4)	78 (85,7)	181 (86,6)	22 (68,8)	21 (95,5)	5 (71,4)	45 (83,3)	33 (76,7)	97 (89,8)	124 (86,1)	132 (85,2)	23 (92)	14 (87,5)	110 (84)	13 (81,3)	10 (90,9)	15 (78,9)	66 (83,5)	145 (87,3)	28 (87,5)	99 (85,3)	205 (85,4)

	Sometimes	2 (4,7)	8 (11,9)	5 (5,7)	23 (14,6)	17 (8,2)	21 (14,1)	5 (10,4)	33 (10,7)	9 (9,9)	23 (11)	6 (18,8)	0 (0)	1 (14,3)	8 (14,8)	5 (11,6)	8 (7,4)	16 (11,1)	16 (10,3)	0 (0)	0 (0)	19 (14,5)	2 (12,5)	1 (9,1)	2 (10,5)	9 (11,4)	14 (8,4)	4 (12,5)	15 (12,9)	23 (9,6)
	Never or almost never	0 (0)	4 (6)	6 (6,8)	4 (2,5)	7 (3,4)	7 (4,7)	5 (10,4)	9 (2,9)	4 (4,4)	5 (2,4)	4 (12,5)	1 (4,5)	1 (14,3)	1 (1,9)	5 (11,6)	3 (2,8)	4 (2,8)	7 (4,5)	2 (8)	2 (12,5)	2 (1,5)	1 (6,3)	0 (0)	2 (10,5)	4 (5,1)	7 (4,2)	0 (0)	2 (1,7)	12 (5)
	P	0,070				0,156		0,045**		0,044**				0,121					0,327						0,663				0,228	
**I feel loved**	Always or almost always	39 (90,7)	59 (88,1)	81 (92)	149 (94,3)	195 (94,2)	133 (89,3)	42 (87,5)	286 (92,9)	81 (89)	194 (92,8)	29 (90,6)	22 (100)	7 (100)	49 (90,7)	34 (79,1)	99 (91,7)	139 (96,5)	140 (90,3)	24 (96)	15 (93,8)	122 (93,1)	14 (87,5)	11 (100)	16 (84,2)	71 (89,9)	156 (94)	30 (93,8)	108 (93,1)	220 (91,7)
	Sometimes	4 (9,3)	7 (10,4)	5 (5,7)	5 (3,2)	9 (4,3)	12 (8,1)	5 (10,4)	16 (5,2)	6 (6,6)	12 (5,7)	3 (9,4)	0 (0)	0 (0)	3 (5,6)	8 (18,6)	6 (5,6)	4 (2,8)	14 (9)	1 (4)	0 (0)	4 (3,1)	2 (12,5)	0 (0)	2 (10,5)	5 (6,3)	8 (4,8)	2 (6,3)	8 (6,9)	13 (5,4)
	Never or almost never	0 (0)	1 (1,5)	2 (2,3)	4 (2,5)	3 (1,4)	4 (2,7)	1 (2,1)	6 (1,9)	4 (4,4)	3 (1,4)	0 (0)	0 (0)	0 (0)	2 (3,7)	1 (2,3)	3 (2,8)	1 (0,7)	1 (0,6)	0 (0)	1 (6,3)	5 (3,8)	0 (0)	0 (0)	1 (5,3)	3 (3,8)	2 (1,2)	0 (0)	0 (0)	7 (2,9)
	P	0,356				0,232		0,358		0,384				0,019**					0,309						0,581				0,158	
**I sleep well**	Always or almost always	20 (46,5)	27 (40,3)	42 (47,7)	94 (59,5)	103 (49,8)	80 (53,7)	20 (41,7)	163 (52,9)	38 (41,8)	115 (55)	15 (46,9)	14 (63,6)	6 (85,7)	34 (63)	18 (41,9)	57 (52,8)	68 (47,2)	69 (44,5)	10 (40)	9 (56,3)	80 (61,1)	9 (56,3)	4 (36,4)	8 (42,1)	43 (54,4)	82 (49,4)	19 (59,4)	55 (47,4)	128 (53,3)
	Sometimes	18 (41,9)	30 (44,8)	24 (27,3)	44 (27,8)	72 (34,8)	44 (29,5)	18 (37,5)	98 (31,8)	43 (47,3)	56 (26,8)	12 (37,5)	5 (22,7)	1 (14,3)	10 (18,5)	11 (25,6)	35 (32,4)	59 (41)	59 (38,1)	8 (32)	5 (31,3)	33 (25,2)	5 (31,3)	6 (54,5)	7 (36,8)	26 (32,9)	55 (33,1)	8 (25)	47 (40,5)	69 (28,7)
	Never or almost never	5 (11,6)	10 (14,9)	22 (25)	20 (12,7)	32 (15,5)	25 (16,8)	10 (20,8)	47 (15,3)	10 (11)	38 (18,2)	5 (15,6)	3 (13,6)	0 (0)	10 (18,5)	14 (32,6)	16 (14,8)	17 (11,8)	27 (17,4)	7 (28)	2 (12,5)	18 (13,7)	2 (12,5)	1 (9,1)	4 (21,1)	10 (12,7)	29 (17,5)	5 (15,6)	14 (12,1)	43 (17,9)
	P	0,013**				0,580		0,326		0,029**				0,004**					0,207						0,841				0,063	
**I have enough leisure time**	Always or almost always	23 (53,5)	26 (38,8)	55 (62,5)	133 (84,2)	130 (62,8)	107 (71,8)	22 (45,8)	215 (69,8)	53 (58,2)	139 (66,5)	25 (78,1)	18 (81,8)	4 (57,1)	46 (85,2)	22 (51,2)	74 (68,5)	91 (63,2)	76 (49)	17 (68)	13 (81,3)	117 (89,3)	8 (50)	4 (36,4)	9 (47,4)	58 (73,4)	111 (66,9)	19 (59,4)	66 (56,9)	171 (71,3)
	Sometimes	16 (37,2)	23 (34,3)	22 (25)	20 (12,7)	54 (26,1)	27 (18,1)	16 (33,3)	65 (21,1)	27 (29,7)	47 (22,5)	6 (18,8)	1 (4,5)	1 (14,3)	6 (11,1)	8 (18,6)	24 (22,2)	42 (29,2)	48 (31)	7 (28)	3 (18,8)	11 (8,4)	5 (31,3)	7 (63,6)	9 (47,4)	14 (17,7)	32 (19,3)	13 (40,6)	31 (26,7)	50 (20,8)
	Never or almost never	4 (9,3)	18 (26,9)	11 (12,5)	5 (3,2)	23 (11,1)	15 (10,1)	10 (20,8)	28 (9,1)	11 (12,1)	23 (11)	1 (3,1)	3 (13,6)	2 (28,6)	2 (3,7)	13 (30,2)	10 (9,3)	11 (7,6)	31 (20)	1 (4)	0 (0)	3 (2,3)	3 (18,8)	0 (0)	1 (5,3)	7 (8,9)	23 (13,9)	0 (0)	19 (16,4)	19 (7,9)
	P	0,000**				0,169		0,003**		0,136				0,000**					0,000**						0,004**				0,012**	

N (column %). Results marked with ** are statistically significant (p<0.05).
