# Supplementary material for: Influence of Sociodemographic Variables on the Lifestyle of the Adult Population: A Multicenter Observational Study
Source: Healthcare (Basel). 2025 Jun 30;13(13):1564. doi: 10.3390/healthcare13131564 (PMC12250193; doi:10.3390/healthcare13131564)
Supplement: Supplementary file 1 [file healthcare-13-01564-s001.zip › Supplementary File S6_ Safety and Unintentional Injuries Dimension.pdf]

Supplementary File S6: Analysis of the frequency and percentage distribution of responses to each item within the Safety and Unintentional Injuries dimension, according to sociodemographic variables.

Variable		Age				Sex		Nationality		Marital status				Level of Education					Occupation						Income				Chronic disease	
Safety and Unintentional Injuries		≤35	36-50	51-65	≥66	Female	Male	Other	Spanish	Single	Married In a relationship	Separated Divorced	Widowed	Illiterate or incomplete Primary Education	Primary Education	Secondary Education	High School or Further Education	University Education	Employed	Self-employed	Unemployed	Retired/Pensioner	Unpaid domestic work	Student	No Income	≤ a 1000	1001-2500	>2501	No	Yes
**Seatbelt, helmet use**	Yes	41 (95,3)	65 (97)	87 (98,9)	155 (98,1)	201 (97,1)	147 (98,7)	300 (97,4)	48 (100)	86 (94,5)	206 (98,6)	32 (100)	22 (100)	7 (100)	53 (98,1)	42 (97,7)	106 (98,1)	140 (97,2)	151 (97,4)	25 (100)	15 (93,8)	128 (97,7)	16 (100)	11 (100)	19 (100)	77 (97,5)	161 (97)	32 (100)	113 (97,4)	235 (97,9)
	Sometimes	1 (2,3)	2 (3)	1 (1,1)	3 (1,9)	5 (2,4)	2 (1,3)	7 (2,3)	0 (0)	4 (4,4)	3 (1,4)	0 (0)	0 (0)	0 (0)	1 (1,9)	1 (2,3)	2 (1,9)	3 (2,1)	3 (1,9)	0 (0)	1 (6,3)	3 (2,3)	0 (0)	0 (0)	0 (0)	2 (2,5)	4 (2,4)	0 (0)	2 (1,7)	5 (2,1)
	No	1 (2,3)	0 (0)	0 (0)	0 (0)	1 (0,5)	0 (0)	1 (0,3)	0 (0)	1 (1,1)	0 (0)	0 (0)	0 (0)	0 (0)	0 (0)	0 (0)	0 (0)	1 (0,7)	1 (0,6)	0 (0)	0 (0)	0 (0)	0 (0)	0 (0)	0 (0)	0 (0)	1 (0,6)	0 (0)	1 (0,9)	0 (0)
	P	0,237				0,536		0,529		0,312				0,990					0,984						0,913				0,346	
**Do not drive under the influence of alcohol or other drugs**	Yes	38 (88,4)	60 (89,6)	83 (94,3)	149 (94,3)	195 (94,2)	135 (90,6)	287 (93,2)	43 (89,6)	80 (87,9)	198 (94,7)	30 (93,8)	20 (90,9)	5 (71,4)	48 (88,9)	41 (95,3)	100 (92,6)	136 (94,4)	143 (92,3)	21 (84)	14 (87,5)	125 (95,4)	15 (93,8)	10 (90,9)	16 (84,2)	71 (89,9)	153 (92,2)	30 (93,8)	108 (93,1)	222 (92,5)
	Sometimes	4 (9,3)	5 (7,5)	4 (4,5)	7 (4,4)	8 (3,9)	12 (8,1)	17 (5,5)	3 (6,3)	8 (8,8)	10 (4,8)	2 (6,3)	0 (0)	1 (14,3)	4 (7,4)	1 (2,3)	8 (7,4)	6 (4,2)	9 (5,8)	3 (12)	1 (6,3)	5 (3,8)	1 (6,3)	1 (9,1)	3 (15,8)	5 (6,3)	10 (6)	2 (6,3)	5 (4,3)	15 (6,3)
	No	1 (2,3)	2 (3)	1 (1,1)	2 (1,3)	4 (1,9)	2 (1,3)	4 (1,3)	2 (4,2)	3 (3,3)	1 (0,5)	0 (0)	2 (9,1)	1 (14,3)	2 (3,7)	1 (2,3)	0 (0)	2 (1,4)	3 (1,9)	1 (4)	1 (6,3)	1 (0,8)	0 (0)	0 (0)	0 (0)	3 (3,8)	3 (18,8)	0 (0)	3 (2,6)	3 (1,3)
	P	0,764				0,222		0,346		0,026**				0,092					0,837						0,555				0,508	
**Respect traffic rules**	Yes	26 (60,5)	53 (79,1)	77 (87,5)	137 (86,7)	174 (84,1)	119 (79,9)	251 (81,5)	42 (87,5)	72 (79,1)	174 (83,3)	24 (75)	21 (95,5)	7 (100)	51 (94,4)	38 (88,4)	87 (80,6)	110 (76,4)	125 (80,6)	14 (56)	14 (87,5)	116 (88,5)	15 (93,8)	7 (63,6)	13 (68,4)	73 (92,4)	139 (83,7)	17 (53,1)	91 (78,4)	202 (84,2)
	Sometimes	15 (34,9)	12 (17,9)	11 (12,5)	20 (12,7)	31 (15)	27 (18,1)	52 (16,9)	6 (12,5)	18 (19,8)	31 (14,8)	8 (25)	1 (4,5)	0 (0)	3 (5,6)	5 (11,6)	18 (16,7)	32 (22,2)	26 (16,8)	11 (44)	2 (12,5)	14 (10,7)	1 (6,3)	4 (36,4)	6 (31,6)	6 (7,6)	23 (13,9)	14 (43,8)	22 (19)	36 (15)
	No	2 (4,7)	2 (3)	0 (0)	1 (0,6)	2 (1)	3 (2)	5 (1,6)	0 (0)	1 (1,1)	4 (1,9)	0 (0)	0 (0)	0 (0)	0 (0)	0 (0)	3 (2,8)	2 (1,4)	4 (2,6)	0 (0)	0 (0)	1 (0,8)	0 (0)	0 (0)	0 (0)	0 (0)	4 (2,4)	1 (3,1)	3 (2,6)	2 (0,8)
	P	0,002**				0,500		0,484		0,392				0,092					0,015**						0,000**				0,250	
**Follow water safety rules**	Yes	39 (90,7)	62 (92,5)	84 (95,5)	157 (99,4)	202 (97,6)	140 (94)	297 (96,4)	45 (93,8)	88 (96,7)	200 (95,7)	30 (93,8)	22 (100)	7 (100)	54 (100)	43 (100)	102 (94,4)	136 (94,4)	144 (92,9)	24 (96)	15 (93,8)	130 (99,2)	16 (100)	11 (100)	19 (100)	76 (96,2)	160 (96,4)	28 (87,5)	108 (93,1)	234 (97,5)
	Sometimes	3 (7)	4 (6)	4 (4,5)	1 (0,6)	3 (1,4)	9 (6)	10 (3,2)	2 (4,2)	2 (2,2)	8 (3,8)	2 (6,3)	0 (0)	0 (0)	0 (0)	0 (0)	6 (5,6)	6 (4,2)	9 (5,8)	1 (4)	1 (6,3)	1 (0,8)	0 (0)	0 (0)	0 (0)	3 (3,8)	4 (2,4)	4 (12,5)	6 (5,2)	6 (2,5)
	No	1 (2,3)	1 (1,5)	0 (0)	0 (0)	2 (1)	0 (0)	1 (0,3)	1 (2,1)	1 (1,1)	1 (0,5)	0 (0)	0 (0)	0 (0)	0 (0)	0 (0)	0 (0)	2 (1,4)	2 (1,3)	0 (0)	0 (0)	0 (0)	0 (0)	0 (0)	0 (0)	0 (0)	2 (1,2)	0 (0)	2 (1,7)	0 (0)
	P	0,060				0,031**		0,298		0,825				0,387					0,641						0,127				0,051	
**Follow home accident prevention recommendations**	Yes	38 (88,4)	60 (89,6)	85 (96,6)	151 (95,6)	191 (92,3)	143 (96)	289 (93,8)	45 (93,8)	85 (93,4)	199 (95,2)	26 (81,3)	22 (100)	7 (100)	53 (98,1)	42 (97,7)	100 (92,6)	132 (91,7)	143 (92,3)	23 (92)	16 (100)	125 (95,4)	15 (93,8)	10 (90,9)	18 (94,7)	78 (98,7)	154 (92,8)	28 (87,5)	106 (91,4)	228 (95)
	Sometimes	4 (9,3)	6 (9)	3 (3,4)	7 (4,4)	15 (7,2)	5 (3,4)	17 (5,5)	3 (6,3)	4 (4,4)	10 (4,8)	6 (18,8)	0 (0)	0 (0)	1 (1,9)	1 (2,3)	8 (7,4)	10 (6,9)	10 (6,5)	2 (8)	0 (0)	6 (4,6)	1 (6,3)	1 (9,1)	1 (5,3)	1 (1,3)	10 (6)	4 (12,5)	9 (7,8)	11 (4,6)
	No	1 (2,3)	1 (1,5)	0 (0)	0 (0)	1 (0,5)	1 (0,7)	2 (0,6)	0 (0)	2 (2,2)	0 (0)	0 (0)	0 (0)	0 (0)	0 (0)	0 (0)	0 (0)	2 (1,4)	2 (1,3)	0 (0)	0 (0)	0 (0)	0 (0)	0 (0)	0 (0)	0 (0)	2 (1,2)	0 (0)	1 (0,9)	1 (0,4)
	P	0,190				0,284		0,839		0,006**				0,549					0,967						0,275				0,409	

N (column %). Results marked with ** are statistically significant (p<0.05).
